# Pore-scale simulation of low-salinity waterflooding in mixed-wet systems: effect of corner flow, surface heterogeneity and kinetics of wettability alteration

**DOI:** 10.1038/s41598-024-56846-0

**Published:** 2024-03-19

**Authors:** Ali Ahmadi-Falavarjani, Hassan Mahani, Shahab Ayatollahi

**Affiliations:** Department of Chemical and Petroleum Engineering, Sharif University of Technology, Tehran, Iran

**Keywords:** Solid Earth sciences, Chemistry, Energy science and technology, Engineering, Physics

## Abstract

The initial wettability state of the candidate oil reservoirs for low-salinity waterflooding (LSWF) is commonly characterized as mixed-wet. In mixed-wet systems, both the two-phase flow dynamics and the salt transport are significantly influenced by the corner flow of the wetting phase. Thus this study aims at comprehensive evaluation of LSWF efficiency by capturing the effect of corner flow and non-uniform wettability distribution. In this regard, direct numerical simulations under capillary-dominated flow regime were performed using the OpenFOAM Computational Fluid Dynamics toolbox. The results indicate that corner flow results in the transport of low-salinity water ahead of the primary fluid front and triggers a transition in the flow regime from a piston-like to multi-directional displacement. This then makes a substantial difference of 22% in the ultimate oil recovery factors between the 2D and quasi-3D models. Furthermore, the interplay of solute transport through corners and wettability alteration kinetics can lead to a new oil trapping mechanism, not reported in the literature, that diminishes LSWF efficiency. While the findings of this study elucidate that LSWF does exhibit improved oil recovery compared to high-salinity waterflooding, the complicating phenomena in mixed-wet systems can significantly affect the efficiency of this method and make it less successful.

## Introduction

In recent years, low-salinity waterflooding (LSWF) has emerged as a promising technique for enhancing oil recovery from oil reservoirs. The method involves manipulating the ionic composition and salinity level of water injected into the reservoir, which alters the wettability state of the reservoir rock and enhances oil displacement^1–4^. LSWF is considered an economically viable and environmentally friendly alternative to traditional enhanced oil recovery methods such as chemical flooding or steam injection, as it requires less energy and fewer chemicals. Despite its potential advantages, there is still much to be understood about the pore-scale mechanisms by which low-salinity water (LSW) works and how it can be optimized for various reservoir types and conditions.

Low-salinity recovery (LSR), observed at the field-scale, marks the final stage of an intricate multi-scale process initiated by the low-salinity effect (LSE) at the atomistic/molecular-scale^5^. However, as noted by Bartels et al.^6^, the occurrence of small-scale effects or LSE alone is inadequate to ensure incremental oil recovery through LSWF. The missing links here are the phenomena at the intermediate scales. In connecting the sub-pore events to field-scale observations, pore-scale, and pore-network scale phenomena play a critically important role among the intermediate scales^3^. For instance, wettability alteration (WA), which has been introduced as the most plausible cause of LSR^2,6–10^, could be observed and measured at the pore-scale. While previous studies have examined the efficiency of LSWF for EOR purposes, few have delved deeper into the pore-scale mechanisms that govern this process. In light of this perspective, pore-scale simulations offer valuable insights into the fundamental physics and intricate interactions occurring at the microscopic level during LSWF. Maes and Geiger^11^ developed a pore-scale numerical model capable of accounting for the effect of surface complexation reactions on the surface potential of carbonate rocks, while also explaining micro-scale experimental findings on LSWF. Aziz et al.^12^ reported that the solute transport in a 2D pore-network is mainly influenced by advection and diffusion, corresponding to flowing and stagnant regions, respectively. This phenomenon may result in heterogeneous WA, and explain lower oil recoveries during tertiary LSWF when compared to secondary injection processes. Additionally, Aziz et al.^13^ examined how the pore size distribution affects tertiary LSWF. Their findings indicated the possibility of additional oil recovery for domains (porous media) exhibiting heterogeneous pore size distribution, attributed to the presence of capillary pressure gradient around the oil ganglia. This observation contrasted with domains characterized by less variation in pore size distribution. Namaee-Ghasemi et al.^14^ investigated the dynamics of tertiary LSWF within an oil-wet pore-doublet model. Lately, Namaee-Ghasemi et al.^15^ expanded their solver to simulate LSWF utilizing a DLVO-based model to calculate the equilibrium contact angle. This mechanistic model captures the LSE more efficiently compared to conventional linear interpolation methods. Despite the attempts to capture a comprehensive picture of the LSE, previous research focused solely on evaluating the LSWF in initially oil-filled 2D models or uniformly wet media, neglecting the significant effects of pore geometry^11–17^. Although Akai et al.^18,19^ conducted LSWF simulations within a 3D pore structure derived from micro-CT images of a mixed-wet carbonate rock, their work did not address the influence of pore geometry on volumetric and displacement sweep efficiencies nor the LSE during LSWF. Pore geometry consisting of surface roughness, angularity, and the pore-size distribution, significantly affects wettability distribution and multiphase flow phenomena such as corner flow occurrence, ultimately exerting a conspicuous impact on multiphase flow dynamics and solute transport through porous media^20,21^.

The flow of water through the corners of a capillary pore channel is commonly referred to as corner flow, which has been a popular topic of study in the literature, with numerous studies dedicated to exploring its nature and characteristics. Zhao et al.^22^ observed that waterflooding in a quasi-2D micromodel with vertical posts resulted in low displacement efficiency under strong imbibition conditions. The low efficiency was attributed to corner flow, where the wetting fluid bypassed pore bodies due to capillary suction in the corners, leading to the entrapment of the defending fluid. Following their work, Hu et al.^23^, using the OpenFOAM toolbox, reported that the length of this wetting fluid (the distance between primary flow front to corner flow tip) is subject to variations based on the capillary number and contact angle. In fact, lower values of these factors would result in faster displacement of the corner flow tip. In another study, Bakhshian et al.^20,21^ observed the corner flow displacement mechanism in both homogeneous and heterogeneous porous media. They found that although corner flow was not controlled by pore geometry, its negative effects were more pronounced in homogeneous porous samples. This is because larger-sized pores within the heterogeneous samples are less likely to host corner flow. Unlike the recent studies^20–23^ in which corner flow takes place under imbibition conditions, drainage can also cause the occurrence of corner flow^24,25^. In this context, Gong and Piri^26^ established a solute transport model employing dynamic pore network modeling (PNM). Through primary drainage and imbibition simulations that incorporated corner flow, they explored the influence of the oil phase on solute transport, within domains exhibiting different residual oil saturations. Their findings indicated a non-monotonic correlation between the dispersion coefficient and water saturation. Additionally, it was found that corner flow affects the heterogeneity of the fluid saturation profile across the network, which in turn influences the extent of dispersive mixing. Despite investigating the impact of corner flow on solute transport, their transport model was not integrated with dynamic two-phase flow simulations and was limited to a water-wetting state, overlooking mixed-wettability. When oil migrates into underground reservoirs that were initially filled with brine, it cannot completely drain angular pores due to the very high capillary pressure required. As a result, water remains in the corners of each pore, while oil occupies the central space. Consequently, organic components of the oil can undergo adsorption or deposition onto the rock surface, leading to a WA over time^27–33^. This WA maintains water-wet conditions in the corners of the angular pores while allocating oil-wetness to the central parts. Considering this aspect, both oil and water can spontaneously imbibe through the pore structure, which best describes the state of mixed-wettability^27^.

Incorporating microscopic heterogeneities, such as surface roughness, into simulation models can further improve the two-phase flow model and more closely mimic the actual reservoir conditions. Surface roughness influences various aspects of flow, including contact angle manipulation and measurements^34^, as well as the control of interface displacement velocity^35^. For instance, Sari et al.^34^ measured the contact angle of an oil droplet on calcite substrates with surface roughness ranging from 17 to 943 nm in the presence of high- and low-salinity brines. The results showed a decrease in contact angle with increasing surface roughness, regardless of the brine type. Zhang et al.^35^ compared flow in smooth and rough capillary channels and found that the excessive drag effect created by surface roughness slows down the displacing fluid relative to a smooth channel. Analyzing rough-walled fractures through a PNM approach, Gong et al.^36^ highlighted that a rougher aperture field induces capillary fingering and can directly impact the oil entrapment via snap-off following an imbibition process. Moreover, it is known that surface roughness affects not only convective observations but also diffusion displacement mechanisms. Pourakaberian et al.^37^ showed that roughness-related tortuosity increases the diffusion time of solutes in a thin water film during LSWF, thereby slowing down WA.

Overall, due to the intricate characteristics of actual porous media, it is uncommon to find uniformly-wet systems in reservoir rocks. Previous research on the corner flow mechanism^20–23^ has primarily focused on examining uniformly-wet porous material, resulting in a limited comprehension of displacement mechanisms under mixed-wettability conditions, particularly during less-understood recovery technologies such as LSWF. Thus, in this paper, we present a mechanistic study investigating the impact of pore geometry and corner flow on the LSE and LSR via direct computational fluid dynamics (CFD) simulations in 2D and 2.5D pore-scale geometries, shedding new light on the underlying physical processes involved, which to our knowledge, were unexplored in the literature.

## Pore-scale simulation methodology

In this study, we utilized OpenFOAM (Open Field Operation and Manipulation) version number 11^38,39^ which is a widely used, free, open-source CFD software from ESI group (https://www.openfoam.com). While other CFD packages prove valuable in fluid dynamics simulations, we found OpenFOAM to be more efficient in simulating fluid–fluid interfaces with faster convergence. OpenFOAM's open-source nature allows for the exploration of novel aspects, especially in cases where there is no pre-existing solver for unprecedented problems. The advantage lies in the cost-free utilization and improvement of OpenFOAM due to its open-source framework. We also employed the interTransportFoam solver, which is well-suited for LSWF studies due to its ability to simulate species transfer. This solver is derived from the interFoam solver of OpenFOAM and belongs to GeoChemFoam, an OpenFOAM-based toolbox (the open-source code available at https://www.julienmaes.com/geochemfoam) that provides a broad spectrum of features to tackle complex fluid flows in multi-scale porous media.

### Governing equations and mathematical modeling

#### Volume of fluid (VOF) method

The VOF approach^40^ provides robust mathematical modeling for simulating two-phase flow systems that involve immiscible and incompressible fluids. It allows for accurate tracking of the interface evolution between different fluids, based on the velocity vector field obtained from the solution of the Navier–Stokes equations. In this method, the location of the interface is determined using an indicator function (*α*), which represents the volume fraction of a particular phase present in a given cell. To calculate fluid properties such as viscosity (*µ*), density (*ρ*), and velocity (**u**), weighted averaging is used between the two fluids involved, where indices 1 and 2 correspond to the water and oil phases, respectively.

1 $$\mu = \alpha {\mu }_{1}+\left(1-\alpha \right){\mu }_{2}$$

2 $$\rho = \alpha {\rho }_{1}+\left(1-\alpha \right){\rho }_{2}$$

3 $$\mathbf{u}= \alpha {\mathbf{u}}_{1}+\left(1-\alpha \right){\mathbf{u}}_{2}$$

Modeling the two-phase flow requires solving the continuity equation (conservation of mass) that is coupled with Navier–Stokes equation (conservation of momentum). These flow equations are given as follows:

Continuity equation:

4 $$\nabla .\mathbf{u}=0$$

Navier–Stokes equation:

5 $$\rho \left(\frac{\partial \mathbf{u}}{\partial t}+\mathbf{u}.\nabla \mathbf{u}\right)=-\nabla p+\nabla .\mathbf{S}+{\mathbf{F}}_{b}+{\mathbf{F}}_{st}$$

where *t* designates time, *p* pressure, $\mathbf{S} =\mu (\nabla \mathbf{u}+ \nabla {\mathbf{u}}^{T})$ viscous stress, ${\mathbf{F}}_{b}$, denotes external body forces, and ${\mathbf{F}}_{st}$ is the interfacial (capillary) forces that act upon the volume occupied by the interface, and is approximated by the continuum surface force (CSF) formulation which is a function of IFT ($\sigma$), interface curvature ($\kappa$), and the indicator function gradient^41^:

6 $${\mathbf{F}}_{st}=\sigma \kappa \nabla \mathrm{\alpha }$$

The CSF method may face limitations due to spurious currents caused by inaccuracies in calculating the interface normal vector ($\mathbf{n}$) and curvature ($\kappa$) at low capillary numbers (${N}_{ca}$)^42^. However, the filtered surface force (FSF) model offers a solution to this problem by reducing spurious currents through smoothing and sharpening techniques^43^. The mean curvature expression is given as follows:

7 $$\kappa =-\nabla \mathbf{n}+\mathbf{n}.\nabla \mathbf{n}.\mathbf{n}$$

Interface normal vector $\left(\mathbf{n}=\frac{\nabla \mathrm{\alpha }}{\left|\nabla \mathrm{\alpha }\right|}\right)$ on the wall can be computed based on the equilibrium contact angle ($\theta$):

8 $$\mathbf{n}={\mathbf{n}}_{s}{\text{cos}}(\theta )+ {\mathbf{t}}_{s}{\text{sin}}(\theta )$$

In which ${\mathbf{n}}_{s}$ and ${\mathbf{t}}_{s}$ are normal and tangential vectors to the rock surface^41^. Finally, the progression of the phase distribution parameter ($\alpha$) is controlled by the following advection equation:

9 $$\frac{\partial \alpha }{\partial t}+\nabla .\left(\alpha \mathbf{u}\right)+\nabla .\left(\alpha (1-\alpha ){\mathbf{u}}_{r}\right)=0$$

where ${\mathbf{u}}_{r}$ is the relative velocity, applied on the interface. To minimize numerical dispersion and achieve a sharper interface, a compression constant (${c}_{\alpha }$) is utilized, whereby ${\mathbf{u}}_{r}$ is subsequently replaced with the compressive velocity^39^:

10 $${\mathbf{u}}_{r}\equiv {\mathbf{u}}_{{\text{comp}}}={\text{min}}\left({c}_{\alpha }{\left|\mathbf{u}\right|}_{{\text{cell}}}, {\text{max}}({\left|\mathbf{u}\right|}_{{\text{domain}}})\right)$$

#### Species transport

Using a single-field format, continuous species transfer (CST) is a substantial technique to simulate species transport in the VOF methodology^44,45^. The global salinity concentration is acquired through the weighted averaging between the two phases:

11 $$c= \alpha {c}_{1}+\left(1-\alpha \right){c}_{2}$$

After using Henry’s law (${c}_{2}= H{c}_{1}$), in which *H *is Henry’s coefficient, Eq. (11) turns into:

12 $$c= {c}_{1}\left[\alpha +H(1-\alpha )\right]$$

By applying the equilibrium-based mean diffusion, suggested by Maes and Soulaine^46^, we have:

13 $$D = \frac{\alpha {D}_{1}+H\left(1-\alpha \right){D}_{2}}{\alpha +H\left(1-\alpha \right)}$$

The CST equation then will be expressed as:

14 $$\frac{\partial c}{\partial t}+\nabla .\mathbf{F}+\nabla .\mathbf{J}=0$$

where $\mathbf{F}$ represents the advective flux, and $\mathbf{J}$ is the diffusive flux. Using the compressive CST (CCST) formulation, the advective flux can be modeled over the interface via compressive velocity^47^:

15 $$\mathbf{F}=c\mathbf{u}+\frac{\left(1-H\right)c}{\alpha +H\left(1-\alpha \right)}\alpha \left(1-\alpha \right){\mathbf{u}}_{{\text{comp}}},$$

16 $$\mathbf{J} = -D\nabla c+D\frac{\left(1-H\right)c}{\alpha +H\left(1-\alpha \right)}\nabla \alpha$$

Assuming that no solute transfer occurs through the oil phase ($H$≈0), Eq. (14) can be rewritten as shown in Eq. (17):

17 $$\frac{\partial c}{\partial t}+\nabla .\left(c\mathbf{u}+c\left(1-\alpha \right){\mathbf{u}}_{{\text{comp}}}\right)+\nabla .\left(-{D}_{1}\nabla c+{D}_{1}c\frac{\nabla \alpha }{\alpha }\right)=0$$

During simulations, a boundary condition (BC) for concentration accounts for the rock-brine reactions on the walls, and is written as^48^:

18 $${D}_{1}^{NS}\nabla c-{D}_{1}^{NS}c\frac{\nabla \alpha }{\alpha }=s$$

Equation (18) elucidates the slow kinetics associated with WA, as reported in our previous studies^2,6,49^, where ${D}^{NS}$ is the near-surface diffusion coefficient at the boundaries, and $s$ signifies the global molar rate of species creation on the surface and can be defined as:

19 $$s= \alpha {s}_{1}+\left(1-\alpha \right){s}_{2}$$

In consideration of the exclusive impact of LSWF on the occurrence of surface reactions, the value of ${s}_{2}$ is assigned as zero. Additionally, since there is no analytical equation to precisely relate the extent of surface reactions with the salinity of the water, it is plausible to postulate that the variable ${s}_{1}$ exhibits a linear gradient, ranging from zero in the presence of high-salinity (${c}^{HS}$), to a maximum value denoted as ${s}_{{\text{max}}}$, under low-salinity (${c}^{LS}$) circumstances:

20 $$s={s}_{{\text{max}}}\frac{{c}^{HS}-{c}_{1}}{{c}^{HS}-{c}^{LS}}$$

Hence, Eq. (18) can be rewritten as follows:

21 $$\nabla c+\left({R}_{s/d}-\frac{\nabla \alpha }{\alpha }\right)c=\alpha {R}_{s/d}{c}^{HS}$$

where ${R}_{s/d}=\frac{{s}_{{\text{max}}}}{{D}_{1}^{NS}\left({c}^{HS}-{c}^{LS}\right)}$ is a constant in which the subscript s/d indicates the ratio of surface reaction over diffusion coefficient. A reasonable value for this parameter was obtained through matching the simulation with experimental results, which is fully explained in the following section.

### Time-scale of LSW-induced wettability alteration

According to Mahani et al.^2^, the slow kinetics of WA is responsible for the slow detachment of oil droplets exposed to LSW from a clay patch or rock surface. Following the study of Namaee-Ghasemi et al.^14,15^ a boundary condition Eq. (21) for concentration accounts for the kinetics of WA and a value of ${R}_{s/d}\approx$5000 m^−1^ can closely match the experimental contact angle data reported by Mahani et al.^2^. Nevertheless, this value was acquired under the stationary conditions of LSW exposure, and depending on numerical capabilities, may require adjustments at higher injection rates (higher ${N}_{ca}$). In fact, by increasing the injection rate to compensate for the time-intensive and prolonged simulations, increasing the rate of LSW-induced WA is not far from being reasonable. Therefore, throughout the pore-scale simulations of LSWF, WA as the main cause of the LSR is evaluated at various rates which provides assorted simulation outcomes. In this regard, simulations are performed with ${R}_{s/d}$ values of 5000 m^−1^ (representing the most delayed LSE), 1000, 100, and 0 m^−1^ (representing instantaneous LSE) to demonstrate the time effect of WA.

### Validation of the CFD model

The solver used in this study for simulating LSWF has been previously validated by Namaee-Ghasemi et al.^14,15^ against two distinct experimental data from Chatzis and Dullien^50^ and Zamula et al.^51^. Furthermore, upon comparing the simulation results with the experimental oil droplet data by Mahani et al.^2^, a good agreement between the two works was found, confirming the model’s capability in describing the LSE. Hence, the same solver is employed in this study, ensuring consistency and reliability in the simulations.

### Wettability alteration modeling

WA stands as a pivotal factor influencing the efficiency of oil recovery in subsurface reservoirs, yet modeling this alteration proves to be a challenging task. Qiao et al.^52^ introduced a reactive transport model that intricately considers competitive surface reactions involving cations, carboxylic groups, and sulfate. Their approach mechanistically connects multiphase displacement, surface complexation, WA, and oil recovery for carbonate rocks. In our study, we uniquely associate WA, represented by contact angle reduction, solely with solute concentration (brine salinity). During LSWF, the contact angle ($\theta$) is computed by interpolation between the contact angle on the oil-wet walls (${\theta }^{HS}$) and a lower contact angle at low-salinity conditions (${\theta }^{LS}$) representing less oil-wetting or more water-wetting states. Our study employs a linear interpolation method, as commonly found in the literature^11–14,19^, where we have utilized a salinity-dependent interpolation factor denoted as $\omega$:

22 $$\theta =\omega {\theta }^{HS}+\left(1-\omega \right){\theta }^{LS}$$

23 $$\omega =\frac{c-{c}^{LS}}{{c}^{HS}-{c}^{LS}}$$

Applying Eq. (23) results in the Full Range Interpolation (FRI) approach for computing the contact angle ($\theta$) in the course of LSWF.

### Pore-scale models and simulation scenarios

#### Geometry and physical properties of pore-scale models

**Figure 1:**
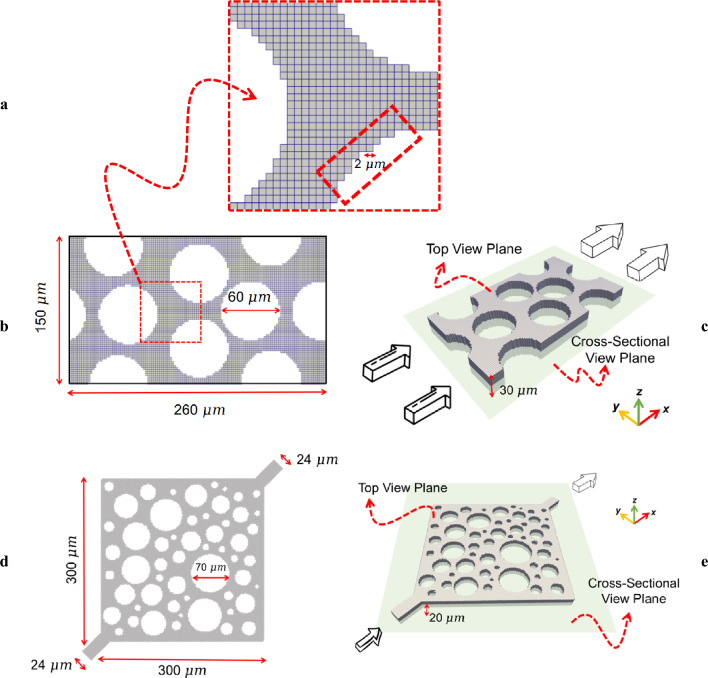
Magnified view of mesh configuration representing the roughness model (**a**), plus the physical dimensions of the homogeneous (**b**, **c**) and heterogeneous (**d**, **e**) geometries and their corresponding meshes. Sub-figures **c** and **e** also showcase the top and cross-sectional view planes, which will be employed to examine the simulation results in detail.

Throughout the simulation of LSWF, corners can act as conduits, creating new pathways for solutes to access areas that may otherwise be inaccessible. To thoroughly investigate this phenomenon, only a pore-network would satisfy the needs. For this purpose, we generated two pore-scale models: one homogeneous and one heterogeneous (see Fig. 1). The homogeneous geometry consists of uniformly sized circular grains in 2D and cylindrical grains (pillars) in 2.5D with mesh cells of size 2 × 2 × 2.5 μm, as shown in Fig. 1 b, c. The heterogeneous geometry, on the other hand, is made up of non-uniform grains with mesh cells sized 2 × 2 × 2 μm, as seen in Fig. 1d, e. To mitigate the high computational costs arising from the increased grid cells in the expanded heterogeneous domain compared to the homogeneous model’s size, the thickness of the heterogeneous geometry was reduced from 30 to 20 $\mathrm{\mu m}$. This adjustment was made to enhance computational efficiency, as each 2.5D simulation of LSWF in the heterogeneous model, even after this adjustment, required approximately 3 weeks run time on a high performance computing system with 64 GB RAM and a 12-processor CPU. However, to maintain numerical accuracy, adjustments were also made to the cell size in the direction of model depth, reducing it from 2.5 to 2 $\mathrm{\mu m}$. While the study by Namaee-Ghasemi et al.^14^ utilized a pore domain with a grid size of 1 $\mathrm{\mu m}$ to capture the diffusive-advective solute transport during LSWF simulations, our heterogeneous geometry, which is at least five times larger, employs grid cells of size 2 $\mathrm{\mu m}$, maintaining the accuracy of the simulations. This ensures that the results are not grid sensitive and numerical dispersion is minimized.

The mesh construction of either geometry was conducted in the *snappyHexMesh* tool of OpenFOAM toolbox, and the roughness model used in our geometries follows the studies of Buchgraber et al.^53^ and Fredriksen et al.^54^, in which vertical grooves are designed to resemble the reservoir-representative surface heterogeneities, applicable to both carbonates^53^ and sandstones^54^. Additionally, surface heterogeneity as roughness was introduced to the model by mesh configuration. As shown in Fig. 1 a*,* the size of this saw-tooth roughness model is dictated by the mesh size.

#### Simulation scenarios

Two sets of simulations were performed to investigate the impact of pore geometry on the LSE/LSR during LSWF. The first set examined the homogeneous pore-network, while the second focused on the heterogeneous pore-network. Identical procedures were used in each set. Initially, oil was injected into the uniformly water-wet pore space until a steady-state condition was achieved.

Subsequently, following the methodology outlined in the study by Landry et al.^55^, the wettability state of the regions associated with the non-wetting phase was altered by creating oil-wet and water-wet patches using Blender, an open-source software (available at https://www.blender.org). This allowed for the creation of mixed-wet pore spaces, which are commonly observed in natural systems. In the literature, several approaches have been utilized to assign a nonuniformly-wet state to a porous medium, incorporating both experimental^56–58^ and numerical^55,59–61^ approaches. The simulations were then continued using secondary LSWF. In this study, and for the heterogeneous geometry, a base case was also established by injecting high-salinity water (HSW) into the pore space at a capillary number of 10^–5^. The purpose of this base case is to provide a benchmark against which the performance of secondary LSWF can be evaluated over different time-scales. The simulation scenarios are summarized in Table 1. Further information on fluid properties and boundary conditions (BCs) is included in the Supplementary Material (SM-1).

**Table 1:** Simulation scenarios performed in this study (simulations marked by a star (*) are presented in the Supplementary Material (SM-5)).

No	Geometry	Dimension	Process	Simulation Objective
1	Homogeneous geometry	2.5D	Oil injection (drainage)	To render a system with mixed-wettability induced by corner flow
Wettability redistribution (rendering a system with mixed-wettability)				
2	Homogeneous geometry	2.5D	Secondary LSWF	To assess the performance of corner flow during LSWF
3	Homogeneous geometry	2D	Oil injection (drainage)*	To render a system with mixed-wettability
Wettability redistribution (rendering a system with mixed-wettability)				
4	Homogeneous geometry	2D	Secondary LSWF	To compare the 2D mode where corner flow is not present with quasi-3D LSWF
5	Heterogeneous geometry	2.5D	Oil injection (drainage)	To render a system with mixed-wettability induced by corner flow
Wettability redistribution (rendering a system with mixed-wettability)				
6	Heterogeneous geometry	2.5D	Secondary HSWF	Employed as a base case to evaluate the performance of LSWF with various WA rates
7			Secondary LSWF (${R}_{s/d}=0$)*	To evaluate the impact of the instantaneous WA rate on LSR affected by corner flow
8			Secondary LSWF (${R}_{s/d}=100$)	To evaluate the impact of various WA rates on LSR affected by corner flow
9			Secondary LSWF (${R}_{s/d}=1000$)	To evaluate the impact of various WA rates on LSR affected by corner flow
10			Secondary LSWF (${R}_{s/d}=5000$)*	To evaluate the impact of the slowest WA rate on LSR affected by corner flow

## Results and discussion

### Simulations performed in the homogeneous geometry

#### Oil migration into porous medium

**Figure 2:**
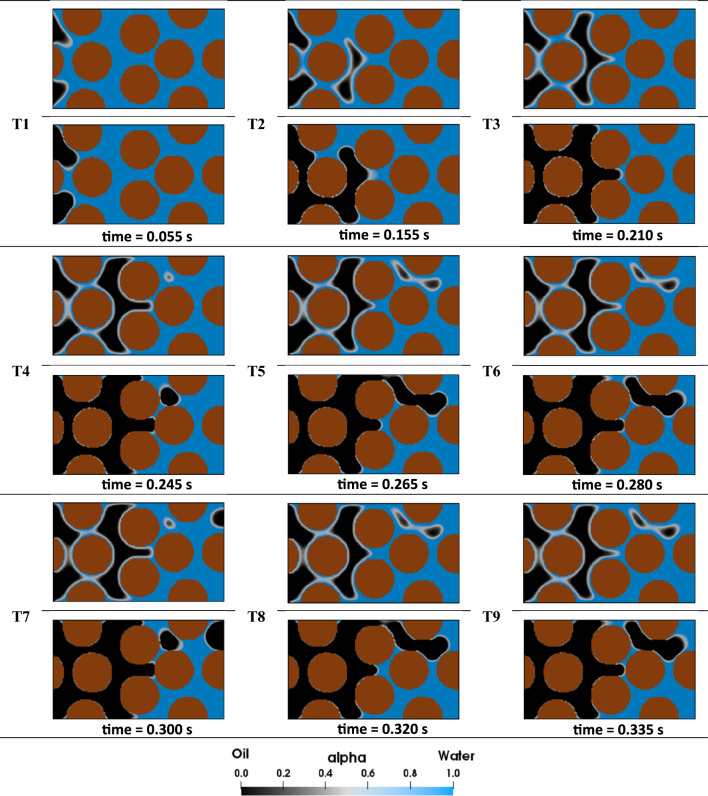
Oil migration into the homogeneous pore-network: In each image set, the upper portion illustrates the top view of the geometry, while the lower portion provides a cross-sectional view of pores to allow for a detailed examination of the displacement. The labels "T1" to "T9" denote the chronological sequence of the pore-scale events.

To replicate the initial fluid distribution in oil reservoirs, oil was injected into pores initially filled with water at a capillary number of 1.33×10^–5^ (see Fig. 2). As the oil moves through the water-wet pores (${\theta }^{IC}=5^\circ$), it encounters high capillary resistance, leading to incomplete drainage of the capillary channels and causing water to remain in the corners. Throughout this process, the oil continues to displace the water until it reaches a steady-state condition (see Fig. 2 T1–T3). The stages illustrated in Fig. 2 T4–T9, indicate that steady-state condition has been established.

#### Effect of corner flow on secondary LSWF

**Figure 3:**
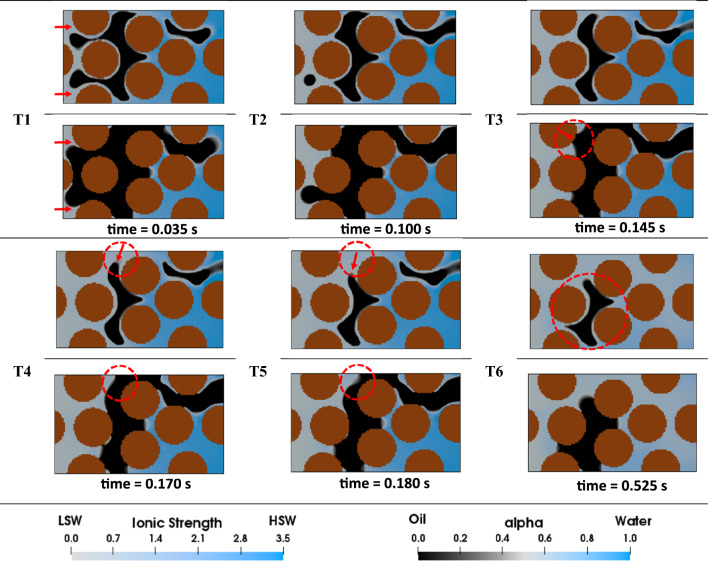
Secondary LSWF into the 2.5D (quasi-3D) homogeneous pore-network. In each image, the upper portion illustrates the top view of the geometry, while the lower portion provides a cross-sectional view of the pores to allow for a detailed examination of the displacement.

After oil migration and modifying the wetting of the porous domain, the simulations were continued by secondary LSWF and using the fluid saturation obtained in Fig. 2 T5 as the initial saturation. The pore-scale displacement results are presented in Fig. 3.

In Fig. 3 T1, T2, it is observed that LSW triggers oil production from left to right at $N_{ca} = 8.33 \times 10^{{{-}6}}$. The contact angle on oil-wet walls decreases from ${\theta }^{HS}=$150^∘^ to ${\theta }^{LS}=$10^∘^ due to LSW action within a time-scale based on the study of Namaee-Ghasemi et al.^14^, which accurately reflects the actual time-scale of LSW WA (${R}_{s/d}=$ 5000). In Fig. 3 T3, the oil remains attached to the oil-wet wall while the LSW bypasses the oil through the water-filled corners of the geometry. This action changes the flow regime and enables LSW to access the pore throat that lies ahead, leading to a reduction in the thickness of the oil layers through WA caused by LSW in the mentioned throat. This thinning process is depicted in Fig. 3 T4, T5. Finally, in Fig. 3 T6, the moveable oil is produced, leaving some trapped oil as identified by the red dashed circle.

**Figure 4:**
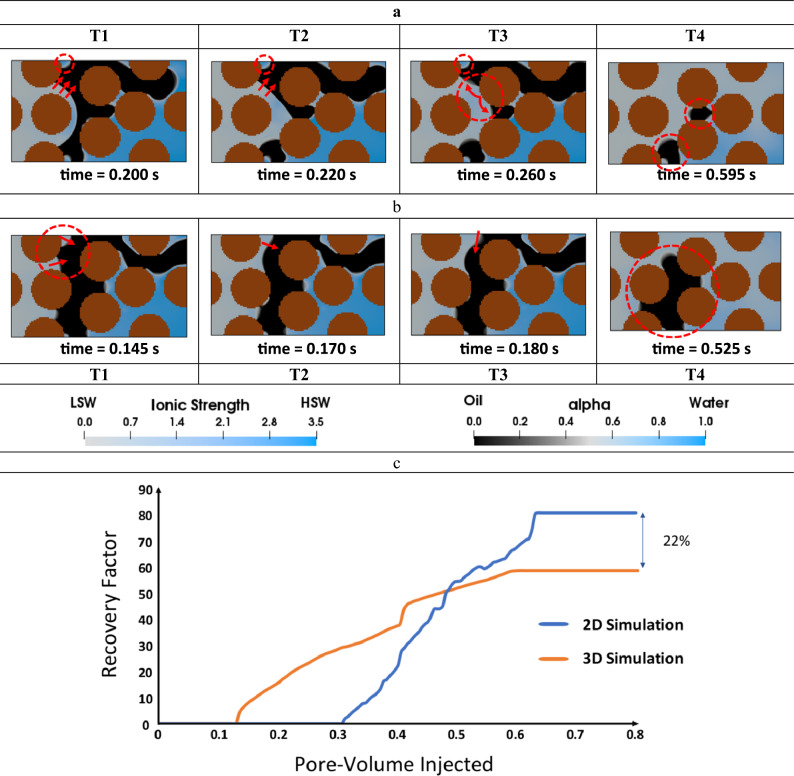
The comparison between the 2D (**a** T1–T4) and cross-sectional view of the quasi-3D (**b** T1–T4) modes of the secondary LSWF simulations into the homogeneous geometry, respectively without and with corner flow. (**c**) Recovery factor curves of the 2D and 2.5D (quasi-3D) LSWF simulations in the homogeneous geometry.

To prevent corner flow from acting as a displacement mechanism during secondary LSWF, simulations were conducted under identical conditions in the 2D model as well (as shown in Table 1). The results of these simulations are presented in Fig. 4. The procedure for the 2D drainage simulation and wettability modification was identical to that used in the 2.5D simulation.

In 2D simulations where the corner flow phenomenon is absent, the contact line of the LSW-oil and consecutively WA is only restricted to the primary front, which results in a piston-like displacement as shown by the red arrows in Fig. 4a T1–T2*.* Additionally, the encircled areas in Fig. 4a T1–T2 confirm that no change in the water saturation can be observed as this area has not been exposed to LSW yet. The displacement then continues until the meniscus collapse takes place upon contact with the grain wall in the red dashed circle in Fig. 4a T3. In contrast, 2.5D simulations show that corners act as conduits, causing multi-directional displacement as shown by red arrows in Fig. 4b T1. The recovery factor (RF) obtained from the 2.5D simulation is 58.3%, while maximum oil recovery of 80.4% is achieved in the 2D case, where LSW remains only behind the primary flow front. Overall, in addition to the observed variation in the flow regime due to corner flow, there is also significant variability in the residual oil saturation as depicted in Fig. 4c. This finding is significant and requires further examination.

In Fig. 3 T1, during the initial stages of imbibition, there exists a considerable distance between the initial oil saturation and the outlet. This distance results in a delay in the onset of the RF curves in both 2.5D and 2D simulations. As fluid flow is not precisely the same in both 2D and 2.5D models, it leads to a slightly distinct fluid distribution after the drainage process. In the 2D simulation, the initial displacement tip of the oil is somewhat more distant from the outlet compared to the 2.5D model at the onset of LSWF simulations, resulting in a separation between the two initiation points in the RF curves. Additionally, due to the presence of oil discontinuity at the outlet and the lack of continuous oil-wet walls throughout the mixed-wet geometry, RF curves no longer follow a linear trend.

In interpreting the results, one could argue that transitioning from 2D to 2.5D models might potentially lead to a reduction in the RF, given that RF reaches 100% within a 1D channel. However, as previously explained, the only theoretically feasible method to exclude the effects of corner flow (derived from a 2.5D framework) is by conducting simulations in a 2D model while keeping other potential aspects (e.g., the shape and size of the geometry) constant compared to a 2.5D model. Additionally, numerous pore-scale phenomena (observed in both homogeneous and heterogeneous models) are directly linked to corner flow, underscoring the importance of this phenomenon.

### Simulations performed in the heterogeneous geometry

To substantiate our findings from the homogeneous geometry, and to conduct a more comprehensive analysis of WA kinetics during LSWF, the following simulations were executed within a (quarter of) five-spot configuration. This conformation not only included greater heterogeneity in grain size but also encompassed an expansion of the entirety of the domain size.

#### Oil migration into porous medium

**Figure 5:**
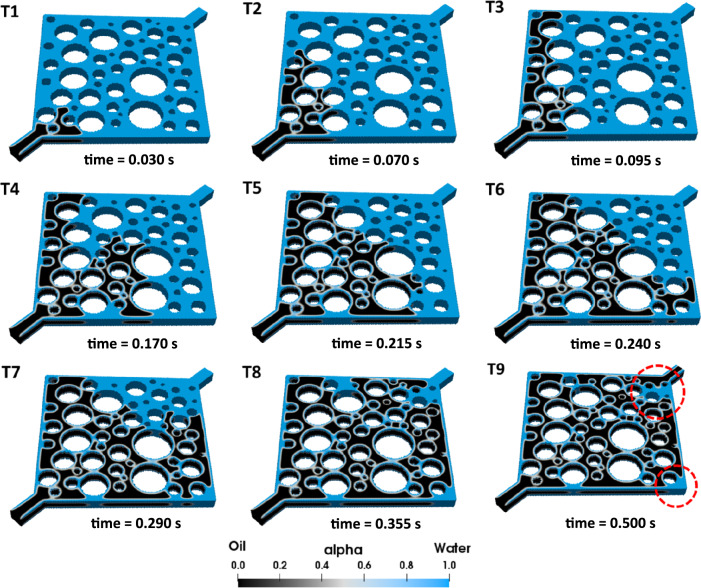
Oil migration into the heterogeneous pore-network. The pore-filling events in this process can be observed through the sub-figures T1 to T9.

At a constant velocity of 0.005 ${\text{m}}/{\text{s}}$, oil migration into the pore space was initiated, corresponding to $N_{ca} = 5 \times 10^{{{-}4}}$. Due to the initially water-wetting state of the pores (${\theta }^{IC}=5^\circ$), the displacing fluid advances preferentially through certain (larger) pores, leaving much of the pore space free of oil at the end of the drainage simulation. Since, this outcome does not necessarily resemble the fluid distribution within a realistic porous material, the injection velocity of oil was set high. The pore-filling sequence of the drainage process simulation is presented in Fig. 5. Once steady-state was reached, the drainage process was stopped, and the final fluid distribution at this stage (Fig. 5 T9) was used as the initial fluid distribution during water injection processes after altering the wettability. As a result, the contact angle on walls adjacent to the oil was changed from ${\theta }^{IC}=5^\circ$ to ${\theta }^{HS}=100^\circ$ to establish mixed-wettability in the domain. Moreover, the water volumes (highlighted with dashed red circles) in Fig. 5 T9, along with the water present in the corners, serve as the initial HSW saturation before LSWF.

#### Effect of corner flow on high-salinity waterflooding (HSWF) efficiency

**Figure 6:**
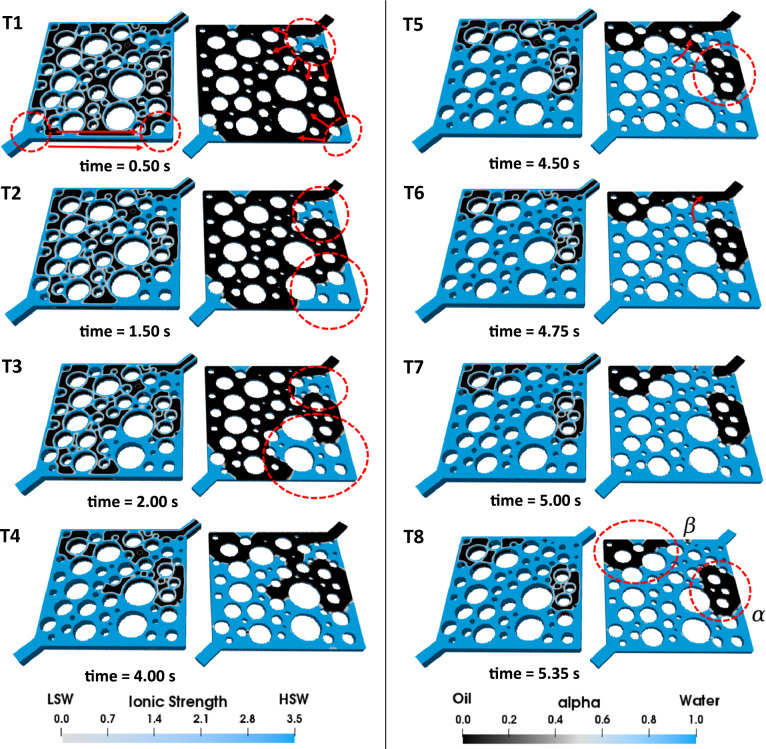
Visualization of HSWF in the heterogeneous pore-network. In each illustration, the left side showcases the complete geometry, while the right side displays a cross-sectional view of the pore structure to emphasize the displacement phenomena at the pore-scale.

During the process of displacing oil by HSW, the entry capillary pressure acts as a barrier to the invading fluid, creating a competitive pathway for the injected HSW to advance through the corners of each pore. In fact, the corner flow displacement mechanism allows water to permeate more easily inside the water-wet and initially water-filled corners than to displace the oil volume inside the center of each pore that is attached to the oil-wet part of the mixed-wet pore walls. As a result of this behavior, the relative permeability of water is enhanced, leading to an expansion of the initial water volume (water banking). Subfigures T1–T3 in Fig. 6 illustrate this process. The injected water supplies the required amount of water to maintain the expansion through the corners of the pores, as shown by the red arrows in Fig. 6 T1.

The expansion of the initial water saturation can be viewed as either favorable or unfavorable, depending on its proximity to the producing outlet. If the initial water volume expands near the outlet, it may obstruct oil production pathways and trap oil, as seen in the red dashed section α in Fig. 6 T8. On the one hand, when the expansion takes place at a distance away from the outlet, it enhances volumetric sweep efficiency and prevents oil entrapment. For example, the failure to produce the trapped oil volume *β* in Fig. 6 T8 is attributed to the oil-wetness of the surrounding walls as well as the bypassing of the oil due to HSW flow through the center of the domain (fingering). Given the symmetry of the geometry, a similar volume of oil is expected to be trapped on the opposite side of the geometry (below the section $\alpha$) due to the same reasons. However, as water can penetrate through the corners of the pores, the initial water saturation in that area expands, preventing the entrapment of this oil cluster.

#### Effect of corner flow on secondary LSWF (${{\varvec{R}}}_{{\varvec{s}}/{\varvec{d}}}=$1000) efficiency

LSW can decrease the equilibrium contact angle in the oil-wet portions of pores with mixed-wettability from ${\theta }^{HS}=$100^∘^ to ${\theta }^{LS}=$10^∘^, corresponding to the ionic strength of 3.5–0.5 mol/L. However, due to the slow kinetics of WA, it is unlikely that the minimum contact angle threshold (${\theta }^{LS}=10^\circ$) is reached during LSWF, especially for locations far from the inlet side of the geometry.

**Figure 7:**
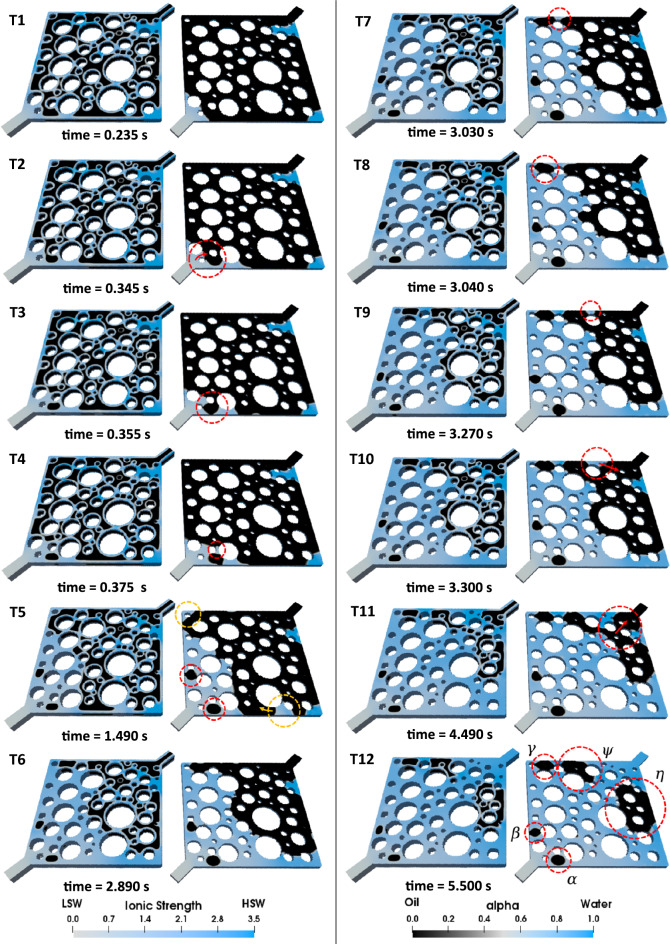
LSWF (*S* = 1000) in the heterogeneous pore-network. In each illustration, the left side showcases the complete geometry, while the right side displays a cross-sectional view of the pore structure to emphasize the displacement phenomena at the pore-scale.

The injection takes place under capillary-dominated conditions where the capillary number is set to 10^–5^. The range of capillary numbers used in this study falls within an accepted domain based on previous pore-scale LSW research^11–13,16–19^. Oil production is initiated as soon as LSW advances through the pore-network (see subfigure T1 in Fig. 7). As demonstrated in Fig. 7 T2, the transition of the LSW front along the red arrow results in the meniscus collapse. At this point, the encircled oil ganglia shown in Fig. 7 T3 remains continuous only through a single throat, making it prone to oil entrapment, which occurs shortly after in the highlighted area in Fig. 7 T4. These observations indicate that oil entrapment in the snap-off form results from the combination of several pore-scale phenomena (that are fully explained in the corresponding section). In the following stages of LSWF, oil is trapped similarly (Fig. 7 T5). However, these phenomena may have positive effects as well. The oil break-up during the displacement of oil by LSW is considered a controlling factor in this regard. In fact, after oil break-up in the yellow dashed areas, oil finds an exit route that is wider than the previous throat in which oil was trapped.

In contrast to HSWF resulting in the trapped oil ganglion *β,* as shown in Fig. 6 T8, LSWF can mobilize this cluster of oil (Fig. 7 T6). However, an oil break-up mechanism prevents part of this relocated oil from being entirely produced (see Fig. 7 T7, T8). This observation is consistent with the findings from Bartels et al.^6^ and Namaee-Ghasemi et al.^14^, who stated that WA is a necessary but not a sufficient condition for observing LSR.

#### Effect of corner flow on secondary LSWF (${R}_{s/d}=100$) efficiency

**Figure 8:**
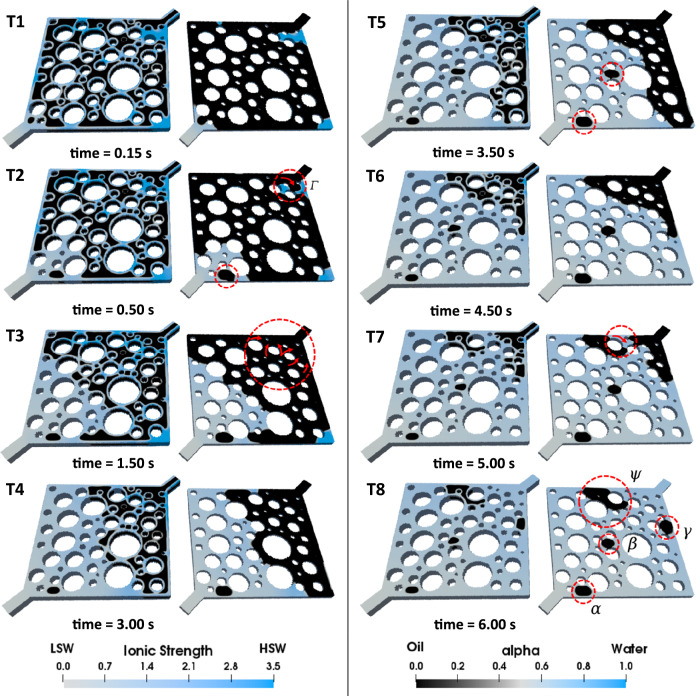
LSWF (*S* = 100) in the heterogeneous pore-network. On the left side of each image, the complete geometry is showcased, while the right side displays a cross-sectional view that enables a thorough analysis of pore-scale displacement phenomena.

To gain additional insights into the time-effect of WA, secondary LSWF simulations were conducted at ${R}_{s/d}=$100, which means that the contact angle reduction upon exposure to LSW takes place more rapidly in comparison with the previous case (${R}_{s/d}=1000$). The results are presented in Fig. 8.

Consequently, the fast reduction in the entry capillary pressure causes LSW to preferentially sweep the oil instead of advancing through the corners of each pore. As a result, the water banking, observed during HSWF, no longer occurs in either of the cases (${R}_{s/d}=100$ or ${R}_{s/d}=1000$) during LSWF. However, due to the faster WA, LSWF at ${R}_{s/d}=100$ can fully displace this water bank (which works as a barrier), creating new flow pathways (see Fig. 8 T2, T3). Subsequently, more oil is recovered at the end of the injection period. As an evidence, the remaining oil volume ( *γ* in Fig. 7 T12) no longer exists in this case.

## Discussion on oil break-up mechanisms and contributing factors affecting overall oil recovery by LSWF

**Figure 9:**
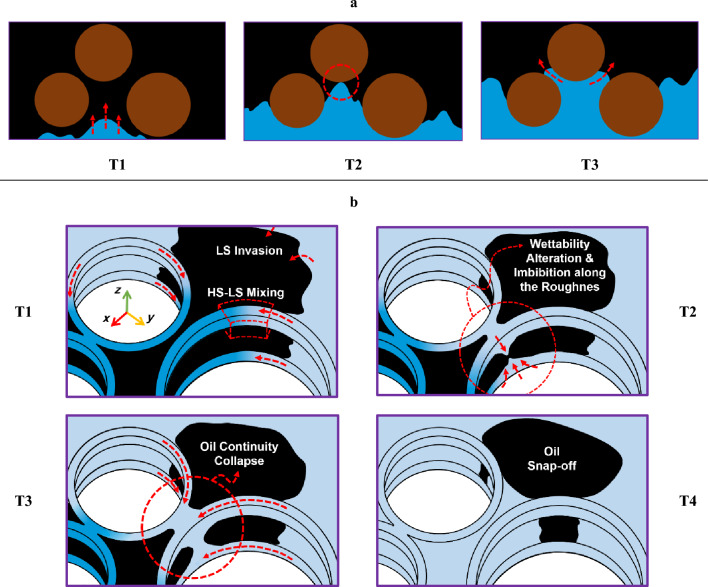
(**a**) Schematic of the I2 mechanism (OBT1), proposed by Lenormand et al.^11^ in a medium with spherical grains; At stage T2, when the water-oil interface reaches the grain walls, it splits into two interfaces that follow separate pathways, as shown at stage T3. The black, blue, and brown colors represent oil, water, and grains respectively. (**b**) 3D schematic illustrating the snap-off mechanism (OBT2); After flowing through the corners (T1), LSW initiates the detachment of oil through WA and begins to imbibe along the vertical roughness at T2. The subsequent process of oil entrapment is shown at T3 and T4. The roughness at the vertical side walls is omitted in the figures to maintain clarity.

Before proceeding with the discussion of the LSWF results, it is essential to elucidate the pore-scale phenomena observed during water injection simulations. Oil ganglia break-up takes place quite often through various mechanisms, as water displaces oil in the porous media. Lenormand et al.^24^ described several processes that lead to discontinuity of the oil phase. For example, the I2 mechanism causes oil rupture when a meniscus comes in contact with the grain surface (or no flow walls), creating two separate interfaces moving through individual pores. This process is prevalent in both 2D and 2.5 (quasi-3D) models of fluid displacement, regardless of the wettability state. In this study, we refer to the I2 mechanism as oil break-up type 1 (OBT1). Schematically, this mechanism occurs inside a geometry similar to our model as illustrated in Fig. 9, subfigure a.

Lenormand et al.^24^ proposed snap-off as another mechanism to explain the oil continuity collapse within water-wet capillary ducts. However, two aspects of this conclusion warrant further consideration. Firstly, mixed-wettability is a more plausible wettability state due to the complex interactions within porous media, than uniformly water-wet state as considered by Lenormand et al.. Secondly, Namaee-Ghasemi et al.^15^ found that during LSWF in oil-wet geometries, snap-off occurs mainly at very high rates of WA, even close to instantaneous WA. Therefore, snap-off is an infrequent occurrence due to the slow wettability change during LSW injection. As a result, our study proposes a new snap-off mechanism, which differs from the one described by Lenormand et al.^24^. This newly observed mechanism is called oil break-up type 2 (OBT2), and it is shown schematically in Fig. 9, subfigure b for a 2.5D model. OBT2 is an important finding because it sheds light on a previously unidentified mechanism that can contribute to the entrapment of oil in the course of LSWF. While displacing the oil, LSW propagation inside angular capillaries develops a primary flow front as well as a corner flow front (Fig. 9 b T1). The latter causes LSW to move ahead of the former (oil–water primary) front, changing the local wettability of rocks. According to the previous studies^14,15^, during LSWF, this wettability change may impose negative effects on LSR if LSW manages to bypass the unswept oil. Unlike the preceding investigations that introduced diffusion through competitive pathways as the only cause of the aforementioned bypassing, corner flow can be considered a new mechanism that provides comparable results.

When LSW enters the corners of the pores it locally alters the wettability of oil-wet walls; whereby water imbibes along the vertical grooves (surface roughness) on the walls (Fig. 9 b T2). The imbibition process finally connects the upper and lower corners of the pore as a consequence of the interplay between corner flow and roughness in the corresponding WA rate (Fig. 9 b T3), providing a path for water layer to swell. The continuous expansion of water layer eventually breaks up the oil, causing trapping of the oil through a snap-off process referred to as OBT2 (Fig. 9 b T4). Entrapment of the oil by the OBT2 mechanism is not always restricted to a single throat. As illustrated in Fig. 7 T9–T11, the disconnection caused by OBT2 can be extended to several pores which brings more obstruction to the current oil production paths and even can be considered as another new entrapment mechanism during LSWF. In essence, conventional snap-off is characteristic of water-wet systems where water moves through the existing water layers on the water-wet walls, pinching off the oil in the pore throat region, and leading to the entrapment of a portion of the oil. On the other hand, OBT2 occurs in mixed-wet systems and is triggered by WA resulting from the exposure of the system to LSW.

Moreover, the fluid flow on rough surfaces observed during OBT2 could be regarded the same as corner flow phenomenon during the imbibition process in the angularities of pores, which was already explained by Hu et al.^23^ and Zhao et al.^22^. This suggests that even within pores with circular cross-sections, the solute transport may still result in a faster oil relocation and/or oil entrapment through the blockage of the oil production path.

Another notable observation is that in comparison to the base case of HSWF, all LSWF cases exhibit a common characteristic of recovering more oil. However, there are factors unique to each LSWF case that may degrade its efficiency. For instance, when ${R}_{s/d}=5000$, the simulation showed a slow rate of reduction in contact angle or WA, which impeded oil mobilization during LSWF and resulted in a displacement pattern similar to that of HSWF after a certain point. The slow WA, however, prevents further oil entrapment caused by the OBT2, promoting increased oil recovery. The Supplementary Material (see Figure S4) provides the numerical simulation sequences for this case.

**Figure 10:**
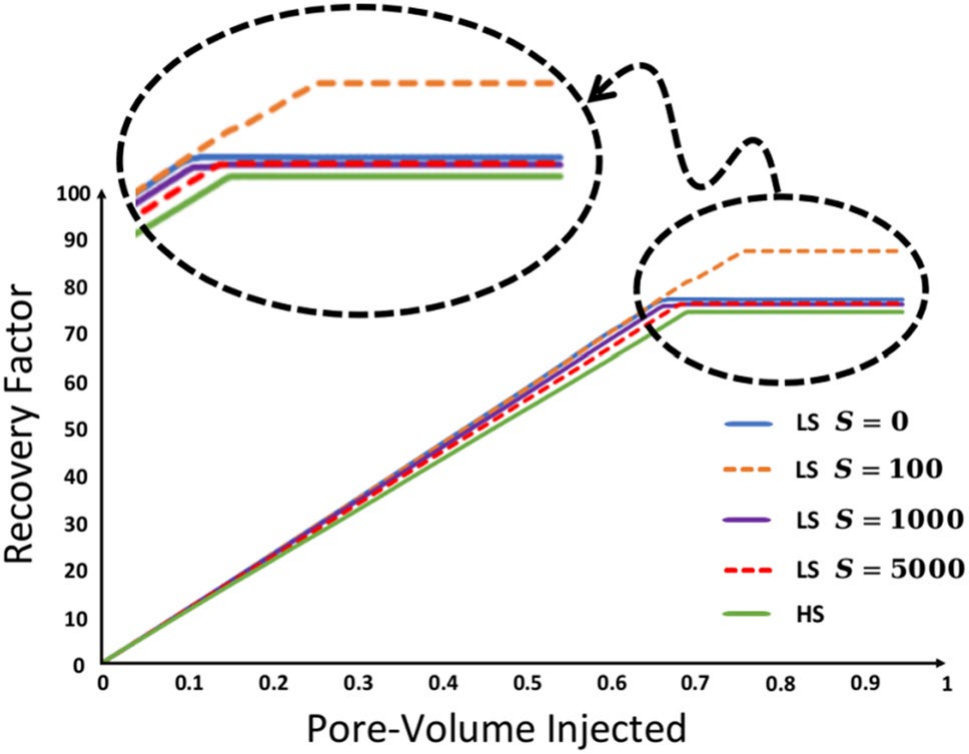
Comparison of the RF curves of LSWF at different WA rates denoted by *S*

In the case where ${R}_{s/d}=1000$, though the oil relocation due to WA is noticeable, numerous oil entrapments caused by geometry-induced OBT2 eventually reduce the RF (Fig. 10). In Fig. 7 T12 multiple trapped clusters of oil are highlighted within dashed circles at the end of the LSWF. Clusters *α*, *β*, and $\gamma$ are repercussions of OBT2, taking place inside a single pore. Trapped oil *ψ* is also left behind due to the same event, however, it is noticeably larger because as explained before, more blocked pores are involved in this event. Despite the current rate of WA, it could not fully displace (drain) the initial water that impeded oil production (water banking), leaving oil volume *η* behind in the pore-network. Regarding the case ${R}_{s/d}=$ 100, snap-off also causes numerous oil entrapments, but the formation of an oil bank is observable in Fig. 8 T4–T6, which accounts for the latest breakthrough (BT) time of this case (see Fig S1 b) corresponding to 0.76 PV. This explains the highest RF among the other cases. Nevertheless, as shown by the red dashed circles in Fig. 8 T8 multiple oil ganglia are trapped. Oil clusters *α*, *β*, and *γ* are consequences of the OBT2 event, and volume *ψ* is incorporated into the trapped oil due to the propagation of the injected LSW toward multiple pores ahead of the primary front. Although LSWF can still provide incremental oil recovery, the adverse effects of corner flow may diminish or erode the LSR even resulting in recoveries as low as that achieved by HSWF. This is quantitatively supported by the RF curves in Fig. 10.

Consistent with the aforementioned discussion, the earliest BT time corresponding to 0.64 PV, belongs to the case ${R}_{s/d}=$0$,$ meaning an instant contact angle decrease from ${\theta }^{HS}=$100^∘^ to ${\theta }^{LS}=$10^∘^. This speeds up oil production inside smaller capillary channels and provides a faster displacement towards the outlet compared to other cases but does not allow sufficient time for the oil to evacuate the pore space adequately. Therefore, based on the RF values in Figure S1 a, oil recovery follows a non-monotonic trend, which means RF does not necessarily increase as the rate of WA is increased. This result aligns with the findings in the existing literature^15,62^. The simulation result of case ${R}_{s/d}=$ 0 is also available in the Supplementary Material (see Figure S5).

The ultimate RF within LSWF cases is represented by the RF values displayed in Figure S1 a. To ensure that no oil remobilization would occur during subsequent pore volumes of LSW injection, the contact angle between water and oil was manually adjusted to ${\theta }^{LS}=$10^∘^, indicating the strong water-wetness of the porous medium. Despite oil detachment from the grain surfaces and walls, no additional oil was recovered, and the residual oil saturation remained unchanged (see Figure S2). Lack of oil remobilization, even though WA occurs, can likely explain some of the unsuccessful practices of LSWF reported in the literature^63^.

## Model limitations

LSR can be influenced by various factors, including reactive transport, reservoir rock heterogeneities, and fluid–fluid interactions resulting from surface-active agents. Despite indirectly addressing some of these effects, such as using a specific boundary condition Eq. (21) to account for surface reaction during LSWF, we intentionally excluded the other phenomena. This simplification allows us to attribute results specifically to the phenomena present in our study, namely mixed-wettability, roughness, and corner flow. Consequently, while corner flow impact is important, it is the net effect of all these factors that ultimately determines the outcome of LSWF.

Moreover, the RF at the field-scale is primarily impacted by permeability variations at reservoir scale and the prevailing flow regimes. Thus, upscaling the findings of this small-scale research to a large-scale problem requires a comprehensive understanding of the link between the micro-scale effects and the macro-scale observations. Nevertheless, exploring the interplay between the effective pore-scale phenomena remains imperative for gaining insights into the outcomes from the macro-scale.

## Conclusions

This study aimed to investigate the performance of LSWF in mixed-wet porous media to assess the combined effects of corner flow and time-effect of WA in rough pore geometries; a key aspect rarely investigated in the literature. Based on the results of pore-scale simulations in different geometries the following conclusions can be made:

- The comparison of 2D and 2.5D simulations with homogeneous grain size highlights the influence of pore geometry, especially corner flow in angular geometries, on two-phase flow characteristics. In the absence of corner flow in 2D simulations, low-salinity water remains confined to the oil–water primary front, exhibiting a piston-like invasion. Conversely, in the 2.5D model with corner flow, multi-directional displacement occurs, enabling low-salinity water to advance ahead of the primary front.
- Corner flow of low-salinity water may offer localized benefits such as a faster wettability alteration and local oil release. However, this may also increase the probability of bypassing in other locations. The study identifies the synchronization of corner flow, roughness, and wettability alteration kinetics leading to the oil snap-off phenomenon through oil break-up type 2 (OBT2) mechanism. This finding suggests that wettability alteration does not necessarily improves oil recovery. When low-salinity water moves beyond the primary flow front via corner flow or dispersion through alternative pathways, wettability alteration occurs ahead of the primary front, impeding oil production. This adverse effect on oil recovery through LSWF underscores how critical it is for low-salinity water to remain within or behind the primary flow region.
- Corner flow not only impacts LSWF efficiency but also influences HSWF outcomes. In HSWF, the entry capillary pressure of mixed-wet pores acts as a barrier, causing injected HSW to bypass oil in the center of the pores and flow into corners. This can lead to the formation of a water bank, acting either as a displacing or blocking mechanism depending on its distance from the outlet. Corner flow has significant implications for the success or failure of both LSWF and HSWF.
- The success of secondary LSWF for EOR hinges on the kinetics of wettability alteration. However, the quantity of recovered oil does not strictly correlate with the extent of local wettability alteration. An optimal rate of wettability alteration is crucial, as there is a risk of water bypassing oil through smaller capillaries, resulting in an early water breakthrough.

While this study mainly examined one specific type of surface roughness, it is recommended to explore the potential effects of other types of roughness on the LSWF efficiency. Additionally, it would be valuable to explore the inclusion of further possible mechanisms such as viscoelasticity or osmosis resulting from the presence of water within angular pores. Finally, as a potential avenue for future exploration, it is recommended to delve more into complex 3D geometries that incorporate primary drainage simulation within a vertical model. This approach would enable the consideration of gravity effects, closely emulating a drainage process representative of reservoir conditions.

### Supplementary Information

Supplementary Information.

## Data Availability

The simulation data generated and analyzed during the current study are available from the corresponding author on reasonable request.

## References

[1] Al-Shalabi EW, Sepehrnoori K (2016). A comprehensive review of low salinity/engineered water injections and their applications in sandstone and carbonate rocks. J. Petrol. Sci. Eng..

[2] Mahani H, Berg S, Ilic D, Bartels W-B, Joekar-Niasar V (2015). Kinetics of low-salinity-flooding effect. SPE J..

[3] Bartels W-B, Mahani H, Berg S, Hassanizadeh SM (2019). Literature review of low salinity waterflooding from a length and time scale perspective. Fuel.

[4] Tang GQ, Morrow NR (1997). Salinity, temperature, oil composition, and oil recovery by waterflooding. SPE Reserv. Eng..

[5] Mahani, H., Thyne, G. Low-salinity (enhanced) waterflooding in carbonate reservoirs. In *Recovery Improvement* 39–107 (Elsevier, 2023).

[6] Bartels W-B, Mahani H, Berg S, Menezes R, van der Hoeven JA, Fadili A (2017). Oil configuration under high-salinity and low-salinity conditions at pore scale: A parametric investigation by use of a single-channel micromodel. SPE J..

[7] Sheng JJ (2014). Critical review of low-salinity waterflooding. J. Petrol. Sci. Eng..

[8] Morrow N, Buckley J (2011). Improved oil recovery by low-salinity waterflooding. J. Petrol. Technol..

[9] Myint PC, Firoozabadi A (2015). Thin liquid films in improved oil recovery from low-salinity brine. Curr. Opin. Colloid Interface Sci..

[10] Tian H, Wang M (2017). Electrokinetic mechanism of wettability alternation at oil-water-rock interface. Surface Sci. Rep..

[11] Maes J, Geiger S (2018). Direct pore-scale reactive transport modelling of dynamic wettability changes induced by surface complexation. Adv. Water Resour..

[12] Aziz R, Joekar-Niasar V, Martínez-Ferrer PJ, Godinez-Brizuela OE, Theodoropoulos C, Mahani H (2019). Novel insights into pore-scale dynamics of wettability alteration during low salinity waterflooding. Sci. Rep..

[13] Aziz R, Niasar V, Erfani H, Martínez-Ferrer PJ (2020). Impact of pore morphology on two-phase flow dynamics under wettability alteration. Fuel.

[14] Namaee-Ghasemi A, Ayatollahi S, Mahani H (2021). Pore-scale simulation of the interplay between wettability, capillary number, and salt dispersion on the efficiency of oil mobilization by low-salinity waterflooding. SPE J..

[15] Namaee Ghasemi, A., Ayatollahi, S., Mahani, H. Insights into the effects of pore structure, time-scale, and injection scenarios on pore-filling sequence and oil recovery by low-salinity waterflooding using a mechanistic DLVO-based pore-scale model. *SPE J.* 2023.

[16] Alizadeh M, Fatemi M (2021). Mechanistic study of the effects of dynamic fluid/fluid and fluid/rock interactions during immiscible displacement of oil in porous media by low salinity water: Direct numerical simulation. J. Mole. Liquids.

[17] Alizadeh M, Fatemi M, Mousavi M (2021). Direct numerical simulation of the effects of fluid/fluid and fluid/rock interactions on the oil displacement by low salinity and high salinity water: Pore-scale occupancy and displacement mechanisms. J. Petrol. Sci. Eng..

[18] Akai T, Alhammadi AM, Blunt MJ, Bijeljic B (2019). Modeling oil recovery in mixed-wet rocks: Pore-scale comparison between experiment and simulation. Trans. Porous Med..

[19] Akai T, Blunt MJ, Bijeljic B (2020). Pore-scale numerical simulation of low salinity water flooding using the lattice Boltzmann method. J. Colloid Interface Sci..

[20] Bakhshian S, Rabbani HS, Hosseini SA, Shokri N (2020). New insights into complex interactions between heterogeneity and wettability influencing two-phase flow in porous media. Geophys. Res. Lett..

[21] Bakhshian S, Rabbani HS, Shokri N (2021). Physics-driven investigation of wettability effects on two-phase flow in natural porous media: Recent advances, new insights, and future perspectives. Trans. Porous Med..

[22] Zhao B, MacMinn CW, Juanes R (2016). Wettability control on multiphase flow in patterned microfluidics. Proc. Natl. Acad. Sci..

[23] Hu R, Wan J, Yang Z, Chen Y-F, Tokunaga T (2018). Wettability and flow rate impacts on immiscible displacement: A theoretical model. Geophys. Res. Lett..

[24] Lenormand R, Zarcone C, Sarr A (1983). Mechanisms of the displacement of one fluid by another in a network of capillary ducts. J. Fluid Mech..

[25] Zhou D, Blunt M, Orr FM (1997). Hydrocarbon drainage along corners of noncircular capillaries. J. Colloid Interface Sci..

[26] Gong Y, Piri M (2020). Pore-to-core upscaling of solute transport under steady-state two-phase flow conditions using dynamic pore network modeling approach. Transp. Porous Med.

[27] Blunt MJ (2017). Multiphase Flow in Permeable Media: A Pore-Scale Perspective.

[28] Anderson WG (1986). Wettability literature survey-part 1: Rock/oil/brine interactions and the effects of core handling on wettability. J. Petrol. Technol..

[29] Salehi M, Johnson SJ, Liang J-T (2008). Mechanistic study of wettability alteration using surfactants with applications in naturally fractured reservoirs. Langmuir.

[30] Basu S, Sharma MM (1997). Characterization of mixed-wettability states in oil reservoirs by atomic force microscopy. SPE J..

[31] Buckley JS, Liu Y, Monsterleet S (1998). Mechanisms of wetting alteration by crude oils. SPE J..

[32] Standnes DC, Austad T (2000). Wettability alteration in chalk: 1. Preparation of core material and oil properties. J. Petrol. Sci. Eng..

[33] Melrose, J. C. Interpretation of mixed wettability states in reservoir rocks. In *SPE Annual Technical Conference and Exhibition* (OnePetro, 1982).

[34] Sari A, Al-Maskari NS, Saeedi A, Xie Q (2020). Impact of surface roughness on wettability of oil-brine-calcite system at sub-pore scale. J. Mole. Liquid..

[35] Zhang L (2021). The effect of surface roughness on immiscible displacement using pore scale simulation. Trans. Porous Med..

[36] Gong Y, Sedghi M, Piri M (2021). Two-phase relative permeability of rough-walled fractures: A dynamic pore-scale modeling of the effects of aperture geometry. Water Resour. Res..

[37] Pourakaberian A, Mahani H, Niasar V (2022). Dynamics of electrostatic interaction and electrodiffusion in a charged thin film with nanoscale physicochemical heterogeneity: Implications for low-salinity waterflooding. Coll. Surfaces A Physicochem. Eng. Aspect.

[38] Weller HG, Tabor G, Jasak H, Fureby C (1998). A tensorial approach to computational continuum mechanics using object-oriented techniques. Comput. Phys..

[39] Rusche, H. Computational fluid dynamics of dispersed two-phase flows at high phase fractions, PhD Thesis, Imperial College London (University of London, 2003).

[40] Hirt CW, Nichols BD (1981). Volume of fluid (VOF) method for the dynamics of free boundaries. J. Comput. Phys..

[41] Brackbill JU, Kothe DB, Zemach C (1992). A continuum method for modeling surface tension. J. Comput. Phys..

[42] Scardovelli R, Zaleski S (1999). Direct numerical simulation of free-surface and interfacial flow. Ann. Rev. Fluid Mech..

[43] Raeini AQ, Blunt MJ, Bijeljic B (2012). Modelling two-phase flow in porous media at the pore scale using the volume-of-fluid method. J. Comput. Phys..

[44] Haroun Y, Legendre D, Raynal L (2010). Volume of fluid method for interfacial reactive mass transfer: Application to stable liquid film. Chem. Eng. Sci..

[45] Marschall H, Hinterberger K, Schüler C, Habla F, Hinrichsen O (2012). Numerical simulation of species transfer across fluid interfaces in free-surface flows using OpenFOAM. Chem. Eng. Sci..

[46] Maes J, Soulaine C (2020). A unified single-field Volume-of-Fluid-based formulation for multi-component interfacial transfer with local volume changes. J. Comput. Phys..

[47] Maes J, Soulaine C (2018). A new compressive scheme to simulate species transfer across fluid interfaces using the volume-of-fluid method. Chem. Eng. Sci..

[48] Graveleau, M. Pore-scale simulation of mass transfer across immiscible interfaces, PhD Thesis, Stanford University Stanford, USA, 2016.

[49] Mohammadi M, Mahani H (2020). Direct insights into the pore-scale mechanism of low-salinity waterflooding in carbonates using a novel calcite microfluidic chip. Fuel.

[50] Chatzis I, Dullien FAL (1983). Dynamic immiscible displacement mechanisms in pore doublets: Theory versus experiment. J. Colloid Interface Sci..

[51] Zamula, Y. S., Batyrshin, E. S., Latypova, R. R., Abramova, O. A., Pityuk, Y. A. Experimental study of the multiphase flow in a pore doublet model. *Journal of Physics: Conference Series* 012052 (IOP Publishing, 2019)

[52] Qiao C, Li L, Johns RT, Xu J (2015). A mechanistic model for wettability alteration by chemically tuned waterflooding in carbonate reservoirs. SPE J..

[53] Buchgraber M, Al-Dossary M, Ross CM, Kovscek AR (2012). Creation of a dual-porosity micromodel for pore-level visualization of multiphase flow. J. Petrol. Sci. Eng..

[54] Fredriksen SB, Rognmo AU, Fernø MA (2018). Pore-scale mechanisms during low salinity waterflooding: Oil mobilization by diffusion and osmosis. J. Petrol. Sci. Eng..

[55] Landry CJ, Karpyn ZT, Ayala O (2014). Relative permeability of homogenous-wet and mixed-wet porous media as determined by pore-scale lattice Boltzmann modeling. Water Resour. Res..

[56] Murison J, Semin B, Baret J-C, Herminghaus S, Schröter M, Brinkmann M (2014). Wetting heterogeneities in porous media control flow dissipation. Phys. Rev. Appl..

[57] Saad AM, Yutkin MP, Radke CJ, Patzek TW (2022). Pore-scale spontaneous imbibition at high advancing contact angles in mixed-wet media: theory and experiment. Energy Fuels.

[58] Song W, Kovscek AR (2015). Functionalization of micromodels with kaolinite for investigation of low salinity oil-recovery processes. Lab Chip.

[59] Zhao J (2018). The effect of wettability heterogeneity on relative permeability of two-phase flow in porous media: A lattice Boltzmann study. Water Resour. Res..

[60] Chen S, Zhang J, Mohammed MZ, Li F, Yan Z, Ding YS (2022). Seepage characteristics of mixed-wettability porous media on the phase-field model. ACS Omega.

[61] Nemer MN, Rao PR, Schaefer L (2021). Coupled influence of wettability alteration and geometry on two-phase flow in porous media. Adv. Water Resour..

[62] Karimpour Khamaneh M, Mahani H (2023). Pore-scale insights into the nonmonotonic effect of injection water salinity on wettability and oil recovery in low-salinity waterflooding. Energy Fuels.

[63] Chávez-Miyauch TE, Lu Y, Firoozabadi A (2020). Low salinity water injection in Berea sandstone: Effect of wettability, interface elasticity, and acid and base functionalities. Fuel.

