# Supplementary material for: Pore-scale simulation of low-salinity waterflooding in mixed-wet systems: effect of corner flow, surface heterogeneity and kinetics of wettability alteration
Source: Sci Rep. 2024 Mar 19;14:6563. doi: 10.1038/s41598-024-56846-0 (PMC10950873; doi:10.1038/s41598-024-56846-0)
Supplement: Supplementary file 1 — Supplementary Information. [file 41598_2024_56846_MOESM1_ESM.docx]

**Supplementary Material**

**Pore-Scale Simulation of Low-Salinity Waterflooding in Mixed-Wet Systems: Effect of Corner Flow, Surface Heterogeneity and Kinetics of Wettability Alteration**

# **Ali Ahmadi-Falavarjani^1^, Hassan Mahani^1,*^, Shahab Ayatollahi^1,*^**

^1^ Department of Chemical and Petroleum Engineering, Sharif University of Technology, Tehran, Iran

^*^ Corresponding authors’ Emails: [hmahani@sharif.edu](mailto:hmahani@sharif.edu); [shahab@sharif.edu](mailto:shahab@sharif.edu)

**SM-1: Fluid properties, initial and boundary conditions (BCs)**

The tables below present the initial and boundary conditions applied to simulate the drainage process and water injection scenarios for both homogeneous and heterogeneous geometries. Additionally, fluid properties are specified in these tables. The values in the tables adhere to the International System of Units (SI).

**Homogeneous geometry:**

**Table S1: The initial condition and boundary conditions of the drainage simulation within the homogeneous geometry**

| **Parameter** | **Initial conditions** | **BCs at inlet** | **BCs at outlet** | **BCs on walls** |
| --- | --- | --- | --- | --- |
| **Indicator function (α)** | $\alpha$ = 1 | $\alpha$ = 0 | $\nabla\alpha$ = 0 | $\alpha$ = $f(\theta)$ |
| **Concentration (c)** | $c$ *=* 3500 | $c$ *=* 0 | $\nabla c$ *=* 0 | $R_{s/d}$ = 3500 |
| **Pressure (p)** | $p$ *=* 0 | $\nabla p$ *=* 0 | $p$ *=* 0 | $\nabla p$ = 0 |
| **Velocity (u)** | $\boldsymbol{u}$ = (0 0 0) | $\boldsymbol{u}$ = (0.0004 0 0) | $\nabla\boldsymbol{u}$ = 0 | $\boldsymbol{u}$ = (0 0 0) |

**Table S2: The initial condition and boundary conditions of the LSWF simulation within the homogeneous geometry**

| **Parameter** | **Initial conditions** | **BCs at inlet** | **BCs at outlet** | **BCs on walls** |
| --- | --- | --- | --- | --- |
| **Indicator function (α)** | The final conditions attained during the drainage process serves as the initial conditions for the subsequent imbibition process. | $\alpha$ = 1 | $\nabla\alpha$ = 0 | $\alpha$ = $f(\theta)$ |
| **Concentration (c)** |  | $c$ *=* 500 | $\nabla c$ *=* 0 | $R_{s/d}$ = 5000 |
| **Pressure (p)** |  | $\nabla p$ *=* 0 | $p$ *=* 0 | $\nabla p$ = 0 |
| **Velocity (u)** | $\boldsymbol{u}$ = (0 0 0) | $\boldsymbol{u}$ = (0.00025 0 0) | $\nabla\boldsymbol{u}$ = 0 | $\boldsymbol{u}$ = (0 0 0) |

**Table S3: Fluid properties of the simulation inside homogeneous geometry**

| **Props.**  **Fluids** | **Density (**$\boldsymbol{\rho}$**)** $\left[ \frac{\boldsymbol{kg}}{\boldsymbol{m}^{\boldsymbol{3}}} \right]$ | **Viscosity (**$\boldsymbol{\mu}$**)** $\left[ \frac{\boldsymbol{kg}}{\boldsymbol{m.s}} \right]$ | **IFT (**$\boldsymbol{\sigma}$**)** $\left[ \frac{\boldsymbol{kg}}{\boldsymbol{s}^{\boldsymbol{2}}} \right]$ | **Diffusion coefficient (**$\mathbf{D}$**)** $\left[ \frac{\boldsymbol{m}^{\boldsymbol{2}}}{\boldsymbol{s}} \right]$ | **Solute concentration (**$\boldsymbol{c}_{\boldsymbol{j}}$**)** $\left[ \frac{\boldsymbol{mole}}{\boldsymbol{m}^{\boldsymbol{3}}} \right]$ | **Contact angle (**$\boldsymbol{^{\circ}}$**)** |
| --- | --- | --- | --- | --- | --- | --- |
| **Oil** | 750 | 0.0015 | 0.03 | 0 | 0 | - |
| **HS water** | 1000 | 0.001 |  | 2e-9 | 3500 | $\theta^{HS}$ =150 |
| **LS water** | 1000 | 0.001 |  |  | 500 | $\theta^{LS}$ =10 |

**Heterogeneous geometry:**

**Table S4: The initial condition and boundary conditions of the drainage simulation within the heterogeneous geometry**

| **Parameter** | **Initial conditions** | **BCs at inlet** | **BCs at outlet** | **BCs on walls** |
| --- | --- | --- | --- | --- |
| **Indicator function (α)** | $\alpha$ = 1 | $\alpha$ = 0 | $\nabla\alpha$ = 0 | $\alpha$ = $f(\theta)$ |
| **Concentration (c)** | $c$ *=* 3500 | $c$ *=* 0 | $\nabla c$ *=* 0 | $R_{s/d}$ = 3500 |
| **Pressure (p)** | $p$ *=* 0 | $\nabla p$ *=* 0 | $p$ *=* 0 | $\nabla p$ = 0 |
| **Velocity (u)** | $\boldsymbol{u}$ = (0 0 0) | $\boldsymbol{u}$ = (0.005 0 0) | $\nabla\boldsymbol{u}$ = 0 | $\boldsymbol{u}$ = (0 0 0) |

**Table S5: The initial condition and boundary conditions of the LSWF simulation within the heterogeneous geometry**

| **Parameter** | **Initial conditions** | **BCs at inlet** | **BCs at outlet** | **BCs on walls** |
| --- | --- | --- | --- | --- |
| **Indicator function (α)** | The final conditions attained during the drainage process serves as the initial conditions for the subsequent imbibition process. | $\alpha$ = 1 | $\nabla\alpha$ = 0 | $\alpha$ = $f(\theta)$ |
| **Concentration (c)** |  | $c$ *=* 500 | $\nabla c$ *=* 0 | $R_{s/d}$ = cont.* |
| **Pressure (p)** |  | $\nabla p$ *=* 0 | $p$ *=* 0 | $\nabla p$ = 0 |
| **Velocity (u)** | $\boldsymbol{u}$ = (0 0 0) | $\boldsymbol{u}$ = (0.0003 0 0) | $\nabla\boldsymbol{u}$ = 0 | $\boldsymbol{u}$ = (0 0 0) |

**Table S6: Fluid properties of the simulation inside heterogeneous geometry**

| **Props.**  **Fluids** | **Density (**$\boldsymbol{\rho}$**)** $\left[ \frac{\boldsymbol{kg}}{\boldsymbol{m}^{\boldsymbol{3}}} \right]$ | **Viscosity (**$\boldsymbol{\mu}$**)** $\left[ \frac{\boldsymbol{kg}}{\boldsymbol{m.s}} \right]$ | **IFT (**$\boldsymbol{\sigma}$**)** $\left[ \frac{\boldsymbol{kg}}{\boldsymbol{s}^{\boldsymbol{2}}} \right]$ | **Diffusion coefficient (**$\mathbf{D}$**)** $\left[ \frac{\boldsymbol{m}^{\boldsymbol{2}}}{\boldsymbol{s}} \right]$ | **Solute concentration (**$\boldsymbol{c}_{\boldsymbol{j}}$**)** $\left[ \frac{\boldsymbol{mole}}{\boldsymbol{m}^{\boldsymbol{3}}} \right]$ | **Contact angle (**$\boldsymbol{\theta}$**)** |
| --- | --- | --- | --- | --- | --- | --- |
| **Oil** | 750 | 0.0015 | 0.03 | 0 | 0 | - |
| **HSW** | 1000 | 0.001 |  | 2e-9 | 3500 | $\theta^{HS}$ = 100 |
| **LSW** | 1000 | 0.001 |  |  | 500 | $\theta^{LS}$ = 10 |

(*) Case-dependent property

**SM-2: Validation**

In this paper, we utilized the same solver as in the previous research conducted by Namaee-Ghasemi et al. [14]. As no additional characteristics were introduced in the solver, it is deemed validated for the current study. The validation of the solver is documented in two parts in Namaee-Ghasemi et al. [14]. The first part validates the solver's accuracy in simulating two-phase behavior during the drainage and imbibition process. The second part confirms the solver's ability to accurately model wettability alteration induced by LSWF.

**SM-3: Diagrams of the oil recovery and water break-through time**

In this section more detailed information regarding the oil recovery by water injection is provided. This information points out to the ultimate oil recovery diagrams as well as the water breakthrough time of each case. These figures can be valuable when analyzing the behaviour of each water injection case.

| **a** | 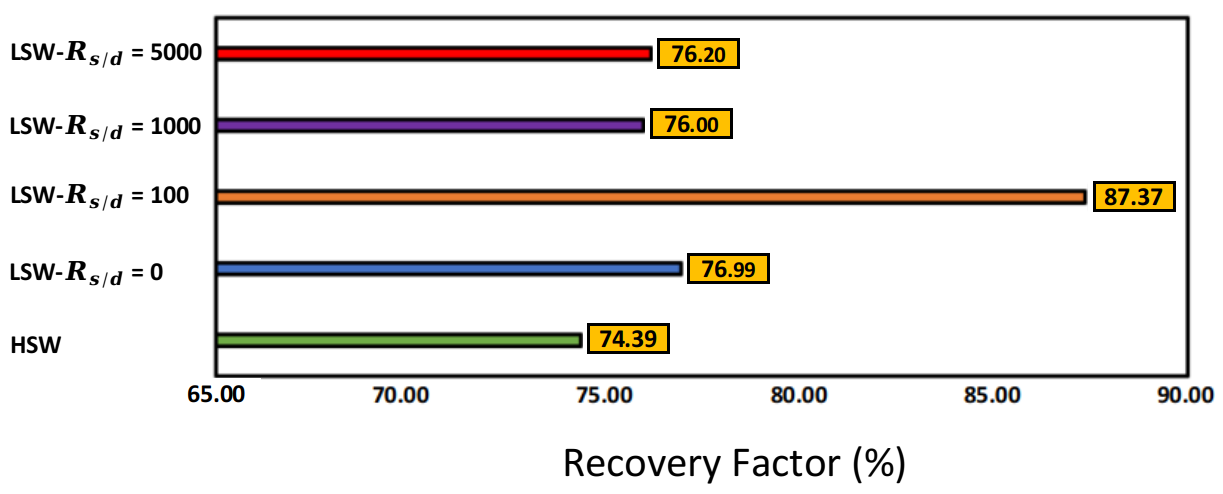 |
| --- | --- |
| **b** | 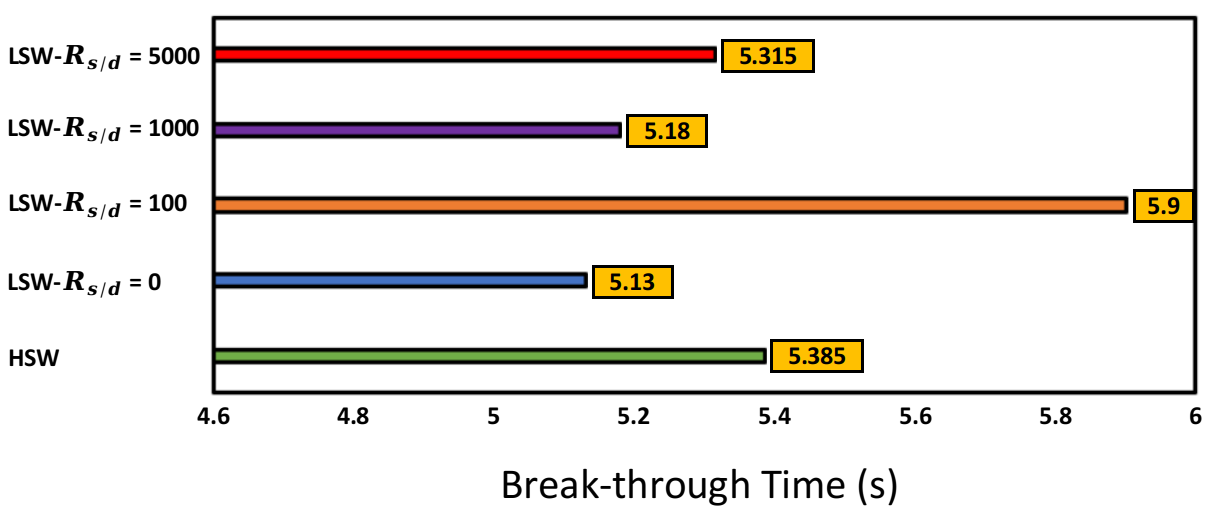 |
| **Fig. S1: Comparison of different WA rates vs. the ultimate RF (a) and BT time (b) of LSWF cases** | |

**SM-4: Oil release test**

Reaching the minimum contact angle ($\theta^{LS}$) during LSWF, due to the slow diffusion of solutes through pore materials, can be very time consuming. To avoid this huge computational cost and to make sure that no more oil can be recovered after one pore volume of LSW injection, the trapped oil at this state was exposed to a brine with a concentration of solute equal to 0.5 liter/mole. This means that the minimum contact angle between oil and LSW ($\theta^{LS}$) is now applied throughout the entire pore geometry. As **Figure S2** depicts, oil is no longer attached to the solid surface, however, the trapped oil cannot be produced either. In other words, when the continuity of oil is disturbed, more water-wetness states of the geometry do not necessarily lead to higher oil recoveries. **Figure S2** illustrates this concept.

| **a** | **b** |
| --- | --- |
| 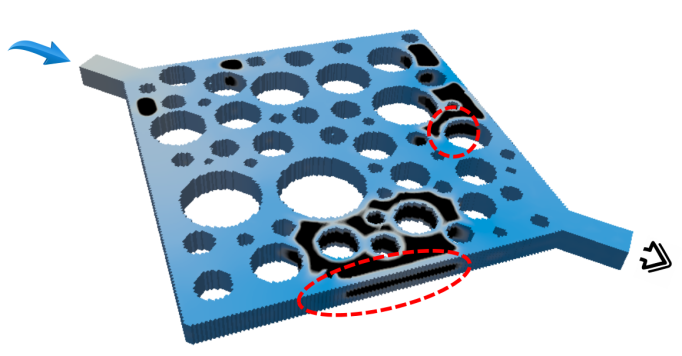 | 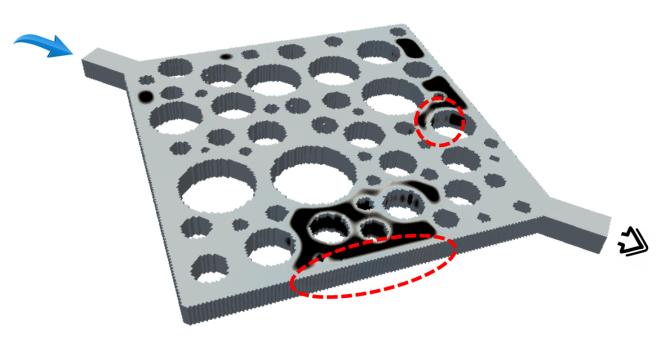 |
| **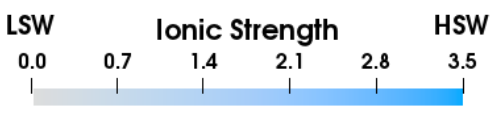** | **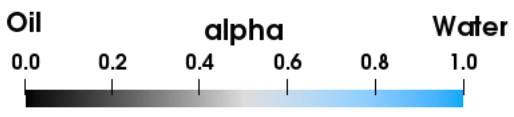** |
| **Figure S2: On the left (a), oil is attached to the geometry walls after almost one pore-volume. On the right (b), the contact angle is manually set to** $\boldsymbol{\theta}^{\boldsymbol{LS}}\boldsymbol{=}$ **10**$\boldsymbol{^{\circ}}$**. Although oil is detached from the previous oil-wet walls, no additional oil recovery is observed in the case** $\boldsymbol{R}_{\boldsymbol{s/d}}\boldsymbol{=}$ **1000.** | |

**SM-5: Pore-scale simulations**

In this section, a detailed visualization of the additional simulations carried out for LSWF is presented.

**2D simulation of drainage process:**

| **T1** | **T2** | | **T3** |
| --- | --- | --- | --- |
| 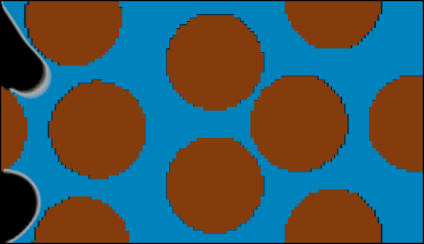 | 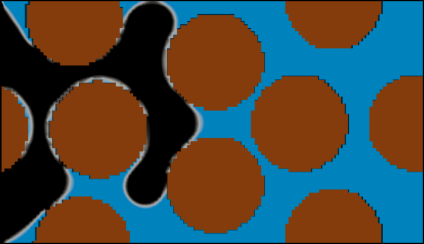 | | 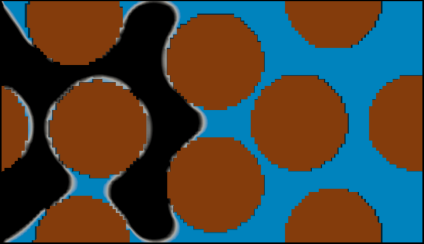 |
| 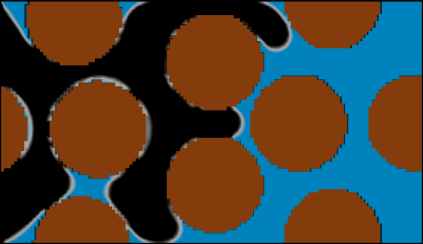 | | 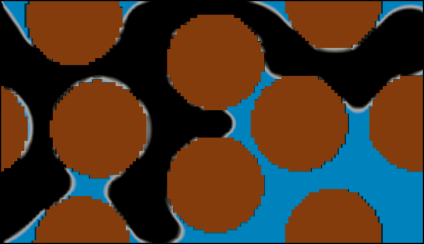 | |
| **T4** | | **T5** | |
| **Figure S3: The simulation of 2D drainage within a homogeneous pore-network was conducted, with the fluid saturation at T4 being chosen for LSWF due to its similarity to the 3D saturation.** | | | |

**Effect of corner flow on secondary LSWF (**$\boldsymbol{R}_{\boldsymbol{s/d}}\boldsymbol{=}$ **5000):**

| **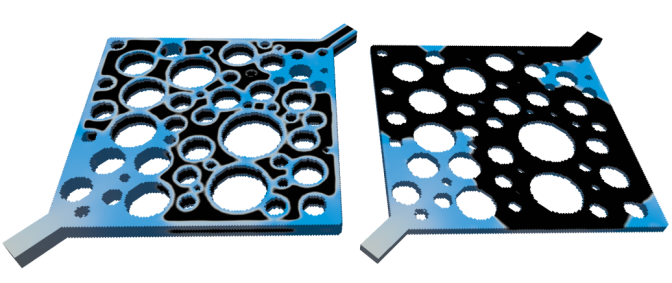**  **T1** | 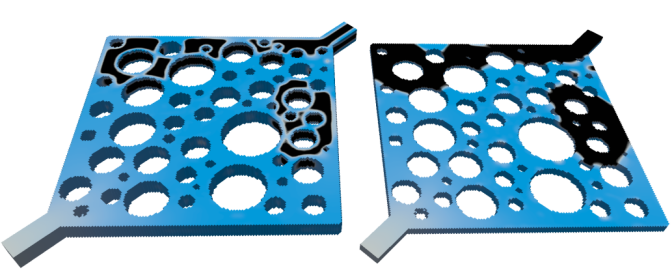  **T4** |
| --- | --- |
| 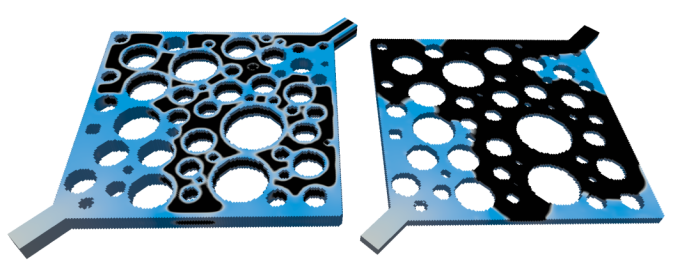  **T2** | 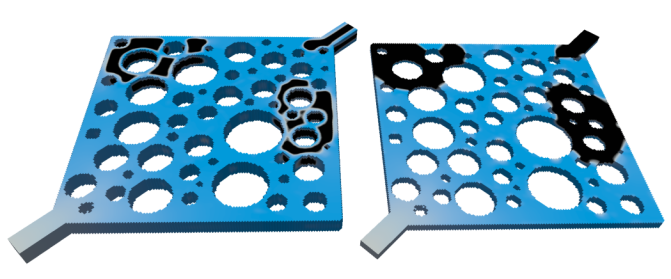  **T5** |
| **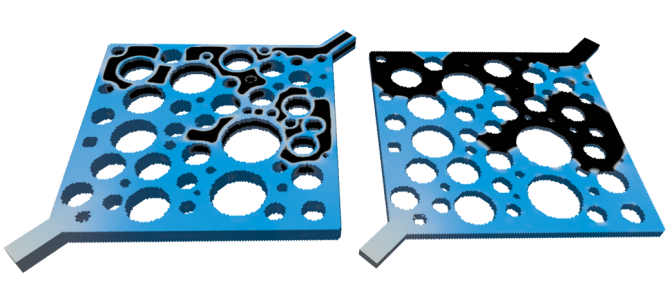**  **T3** | 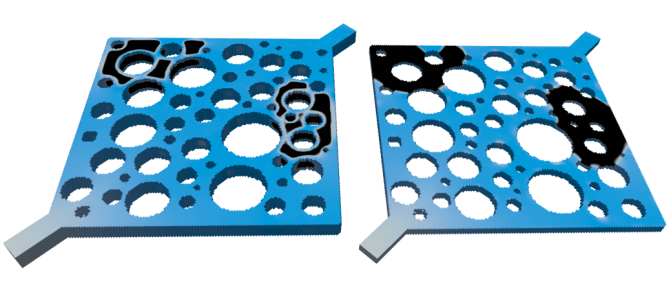  **T6** |
| **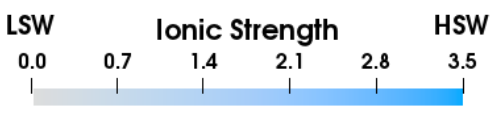** | **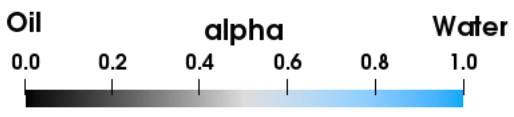** |
| **Figure S4: LSWF (**$\boldsymbol{R}_{\boldsymbol{s/d}}\boldsymbol{=}$ **5000) into heterogeneous pore-network. On the left side of each image, the complete geometry is showcased, while the right side displays a cross-sectional view that enables a thorough analysis of pore-scale displacement phenomena.** | |

**Effect of corner flow on secondary LSWF (**$\boldsymbol{R}_{\boldsymbol{s/d}}\boldsymbol{=}$ **0):**

| **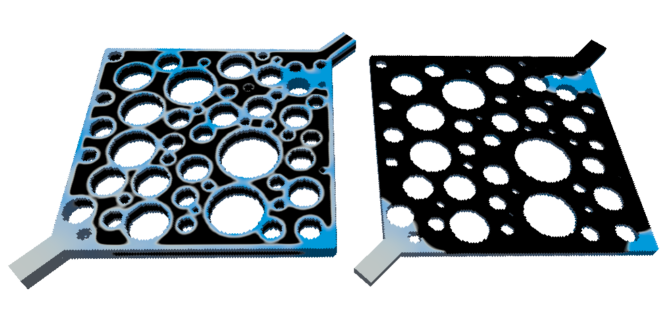**  **T1** | **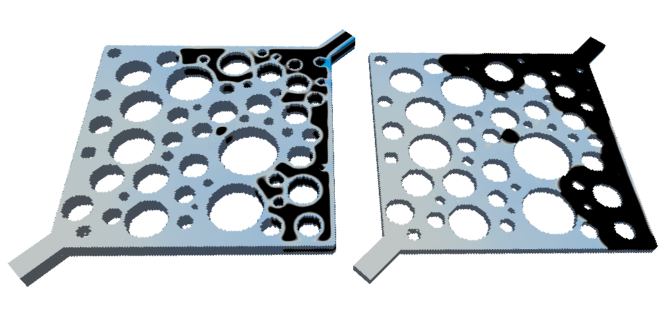**  **T4** |
| --- | --- |
| **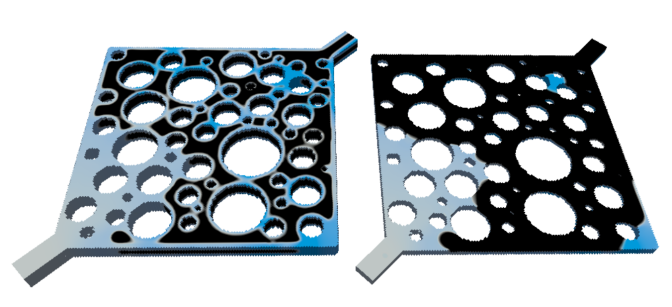**  **T2** | **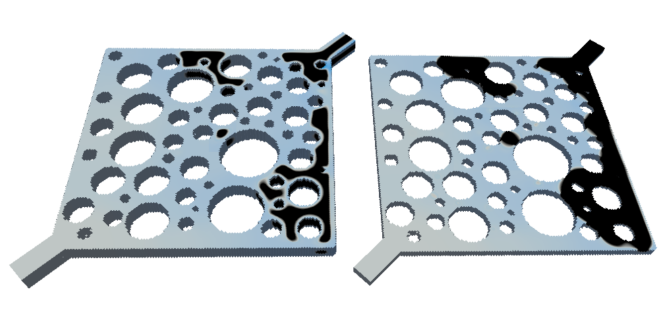**  **T5** |
| **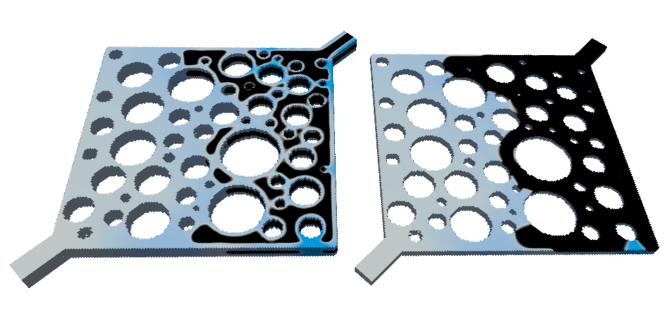**  **T3** | **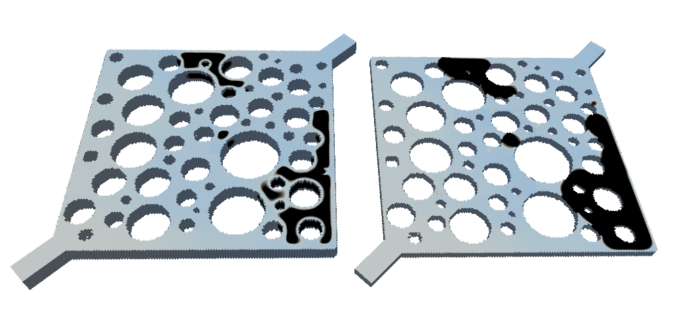**  **T6** |
| **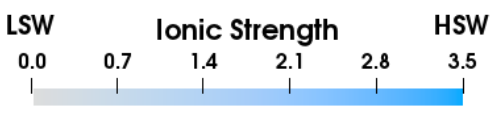** | **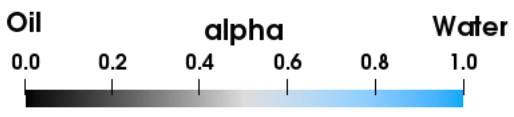** |
| **Figure S5: LSWF (**$\boldsymbol{R}_{\boldsymbol{s/d}}\boldsymbol{=}$ **0) into heterogeneous pore-network. On the left side of each image, the complete geometry is showcased, while the right side displays a cross-sectional view that enables a thorough analysis of pore-scale displacement phenomena.** | |
